# *GJA1* (connexin43) is a key regulator of Alzheimer’s disease pathogenesis

**DOI:** 10.1186/s40478-018-0642-x

**Published:** 2018-12-21

**Authors:** Yuji Kajiwara, Erming Wang, Minghui Wang, Wun Chey Sin, Kristen J. Brennand, Eric Schadt, Christian C. Naus, Joseph Buxbaum, Bin Zhang

**Affiliations:** 1 Department of Psychiatry, Icahn School of Medicine at Mount Sinai, New York, NY 10029 USA; 2 Department of Genetics and Genomic Sciences, Icahn School of Medicine at Mount Sinai, New York, NY 10029 USA; 3 Mount Sinai Center for Transformative Disease Modeling, Icahn Institute of Genomics and Multiscale Biology, Icahn School of Medicine at Mount Sinai, New York, NY 10029 USA; 4 Department of Cellular and Physiological Sciences, University of British Columbia, Vancouver, British Columbia V6T 1Z3 Canada; 5 Department of Neuroscience, Icahn School of Medicine at Mount Sinai, New York, NY 10029 USA; 6 Friedman Brain Institute, Icahn School of Medicine at Mount Sinai, New York, NY 10029 USA; 7 Current address: Denali Therapeutics,, South San Francisco,, CA 94080 USA

**Keywords:** Alzheimer’s disease, GJA1, Cx43, connexin43, Gene networks, amyloidβ, Astrocyte

## Abstract

*GJA1* (connexin43) has been predicted as the top key driver of an astrocyte enriched subnetwork associated with Alzheimer’s disease (AD). In this study, we comprehensively examined *GJA1* expression across 29 transcriptomic and proteomic datasets from post-mortem AD and normal control brains. We demonstrated that *GJA1* was strongly associated with AD amyloid and tau pathologies and cognitive functions. RNA sequencing analysis of *Gja1*−/− astrocytes validated that *Gja1* regulated the subnetwork identified in AD, and many genes involved in Aβ metabolism. Astrocytes lacking *Gja1* showed reduced Apoe protein levels as well as impaired Aβ phagocytosis. Consistent with this, wildtype neurons co-cultured with *Gja1*−/− astrocytes contained higher levels of Aβ species than those with wildtype astrocytes. Moreover, *Gja1*−/− astrocytes was more neuroprotective under Aβ stress. Our results underscore the importance of *GJA1* in AD pathogenesis and its potential for further investigation as a promising pharmacological target in AD.

**Electronic supplementary material:**

The online version of this article (10.1186/s40478-018-0642-x) contains supplementary material, which is available to authorized users.

## Introduction

Alzheimer’s disease (AD) reflects multifactorial genetic and environmental perturbations that, in turn, cause pleiotropic changes in molecular networks linking a host of biological processes. By employing an integrative network biology approach to analyze a large-scale genetic and gene expression dataset in late onset Alzheimer’s disease (LOAD), we previously conducted an unbiased identification and prioritization of gene networks associated with clinical and pathological progression of the disease [98]. In addition to a prominent driver of LOAD, an immune response subnetwork (module) governed by *TYROBP*, we identified other network modules within which gene coexpression patterns were significantly changed in LOAD subjects compared to controls. In the current study, we focused on the identification and characterization of causal regulators of a module that shows a dramatic loss of coordination in AD and that includes *APOE*, a well-known risk factor of AD [18, 82]. This module was highly enriched in astrocyte specific genes, with *GJA1* predicted as its top regulator.

*GJA1*, also known as connexin43 (Cx43), is a member of the connexin family of proteins that is highly conserved among vertebrates. Members of the connexin family exist at the plasma membrane as hexameric complexes known as connexons, and function as connexin hemichannels allowing permeability to small molecules and ions [28]. Two connexons at the apposed cell surface of adjacent cells form a trans-dimer called a gap junction channel (GJC). Gap junctions are clusters of gap junction channels, and mediate efficient and rapid bidirectional inter-cellular transmission and transport of electrical and chemical signals [55, 80]. Cx43 also functions as a unitary channel (hemichannel) to participate in paracrine communication [17], but this activity is often associated with pathological conditions [21]. *GJA1* and *GJB6* (connexin30) are predominantly expressed in mature astrocytes [27, 93, 99], forming astrocytic networks facilitating propagation of calcium waves, potassium and glutamate buffering, and metabolic coupling [66, 70, 77, 78, 83, 89]. Astrocytic gap junctions are critical for neuronal function, as evidenced by the profound neurological phenotypes in *Gja1*/*Gjb6* double knockout mice [56].

Numerous autosomal dominant *GJA1* mutations have been reported to cause oculodentodigital dysplasia (ODDD), which is characterized by craniofacial and limb dysmorphisms [42]. Patients with ODDD frequently manifest neurological symptoms including paraparesis, white matter abnormalities, hearing and vision loss, and low IQ [29, 54]. *GJA1* mutations associated with ODDD are mostly loss-of-function, although a few gain-of-function cases have also been identified, indicating that diverse functional perturbations of *GJA1* can lead to ODDD [42].

Although mice deficient in astrocytic *Gja1* are grossly normal, they show reduced synaptic plasticity [30], increased propagation of synaptic depolarization and neuronal inactivation (“spreading depression”) [84] and impaired in rotarod performance [26]. Mice deficient in both *Gja1* and *Gjb6* spontaneously develop pathological myelin degeneration without overt neuronal atrophy [56]. Loss or reduced expression of *Gja1* leads to enlarged infarct size upon induced-ischemia, and the loss of only the C-terminal tail of Cx43 appeared to mediate the damaging effects [41, 44, 51, 64, 65, 85]. These studies indicated that *Gja1* plays a neuroprotective function and restricts neuronal damage under oxidative and metabolic stress.

*GJA1* has been previously investigated in the context of Alzheimer’s disease. GJA1 (Cx43) immunoreactivity was found to be enriched in astrocytes surrounding amyloid plaques in post-mortem AD brain [63] and a APP/PS1 mouse model [59] and Cx43 hemichannel activity was increased in the APP/PS1 mouse model [96]. Cx43 deficiency or pharmacological blockade of connexins in APP/PS1 mice appeared to reduce dystrophic neurites, mitochondrial oxidative stress, and cognitive impairment without altering amyloid pathology [73, 96, 97].

Here we corroborated the evidence of *GJA1* dysregulation in AD by analyzing *GJA1* expression in a large number of transcriptomic and proteomic datasets from pathologically and clinically characterized LOAD brain samples. We showed that a number of known AD risk factor genes were significantly correlated with *Gja1* in multiple brain regions in AD. We constructed and validated *GJA1* regulated gene networks in AD. We revealed that *Gja1* regulated the expression of more than half of the known AD risk factor genes. We further demonstrated the effect of *Gja1*-deficiency on astrocyte function and the subsequent impact on co-cultured neurons.

## Materials and methods

### Data preprocessing

Three clinical cohorts of post-mortem brain samples from patients with Alzheimer’s disease (AD) symptoms of various severity, and normal controls were subject to RNA microarray and/or RNA-seq analysis to detect changes in mRNA expression caused by AD pathology. As summarized in (64), RNA microarray assays were performed on RNAs extracted from prefrontal cortex (PFC), visual cortex (VC), and cerebellum (CB) of post-mortem brain cortex tissues in the Harvard Brain Tissue Bank (HBTRC) [98]. Details of the research such as neuropathological phenotypic traits of AD subjects and normal controls, RNA microarray assays, data preprocessing and covariate adjustment were described in [98]. Both RNA microarray and RNA-seq assays were conducted using RNAs collected from post-mortem brains in the Mount Sinai Brain Bank (MSBB) cohort [31, 91]. Experimental design and data analysis of RNA microarrays on 19 distinct brain regions of post-mortem brain tissues in the MSBB cohort were presented by Wang et al. [91]. Single-end RNA-seq assays were performed on RNAs extracted from four selected regions (BM10, frontal pole (FP); BM22, superior temporal gyrus (STG); BM36, parahippocampal gyrus (PHG); BM44, inferior frontal gyrus (IFG)) of post-mortem brain tissues in the MSBB cohort. The raw sequence reads were aligned to human genome hg19 with the star aligner (v2.3.0e) [22]. Then the gene level expression was quantified by featureCounts (v1.4.4) [49] based on Ensemble gene model GRCh37.70. The gene level read counts data was normalized using the trimmed mean of M-values normalization (TMM) [76] method to adjust for sequencing library size difference. The normalized data was further adjusted for the covariates postmortem interval (PMI), race, RNA integrity number (RIN), gender, rate of exonic reads, and batch using a linear mixed model [33], where batch was treated as a random effect. The residuals from the regression model were used for downstream analysis. In addition, proteomics assays were performed on proteins extracted from the BM10 region of post-mortem brains in the MSBB cohort, and raw counts summarized at the gene/protein level were provided by the Genomics Core at the Icahn School of Medicine at Mount Sinai. These raw counts were further corrected by covariates (PMI + AOD + batch + gender) using a linear model described by Wang et al. [91], and the residuals after correction were used for further downstream analysis.

The Religious Orders Study and Memory and Aging Project (ROSMAP) cohort consists of ROS and MAP studies [7]. The detailed information regarding ROSMAP study was described in previous studies [8, 9]. Normalized RNA-seq expression data were downloaded from the AMP-AD Knowledge Portal at Synapse upon authentication by the AMP-AD Consortium (doi:10.7303/syn3388564). Genes with at least 1 FPKM in at least 10% of the samples were selected and then the data was corrected for confounding factors including batch, PMI, gender and RIN via a linear model. The residuals after correction were used for further downstream analysis.

### Bayesian causal network analysis

Bayesian causal network was constructed by integrating genome-wide gene expression, SNP genotype, and known transcription factor (TF)-target relationships. Briefly, we first computed expression quantitative trait loci (eQTLs) and then employed a formal statistical causal inference test (CIT) [62] to infer the causal probability between gene pairs associated with the same eQTL. The causal relationships inferred were used, together with TF-target relationships from the ENCODE project, as structural priors for building a causal gene regulatory network from the gene expression data through a Monte Carlo Markov Chain (MCMC) simulation based procedure [100]. We followed a network averaging strategy in which 1000 networks were generated from the MCMC procedure starting with different random structure, and links that shared by more than 30% of the networks were used to define a final consensus network structure. To ensure the consensus network is a directed acyclic graph, an iterative de-loop procedure was conducted, removing the most-weakly supported link of all links involved in any loop. Following Zhang et al. [98], we performed Key Driver Analysis (KDA) on the consensus Bayesian network to identify key hub genes which regulated many downstream nodes.

### RNA sequencing and data processing

Total RNAs were sequenced via the Illumina HiSeq 2500 system with 100 nt paired-end read. Sequencing reads were aligned to mouse reference genome mm10 (GRCm38.75) STAR aligner [22] guided by UCSC gene model. Accepted mapped reads were summarized to gene levels using the featureCounts [49] program. Raw count data were normalized by the voom function in the R limma package [75], and then differential expression was called by the moderated t-test implemented in limma. Differentially expressed genes (DEGs) were defined to have at least 1.2-fold change in expression and BH-adjusted *p* < 0.05 for two comparisons including *Gja1*−/− astrocytes versus wildtype astrocytes and coculture of *Gja1*−/− astrocytes and neurons versus co-culture of wildtype astrocytes and neurons. The raw and processed data are available at doi: 10.7303/syn11711769.

### Construction of *GJA1* centric co-expression networks

We constructed *GJA1* centric consensus co-expression networks from 8 cohort datasets from three cohorts including MSBB (4 brain regions), ROSMAP (1 brain region) and HBTRC (3 brain regions). In each dataset, the genes significantly correlated with *GJA1* were identified based on BH-corrected *p* value < 0.05. From those significant correlations, a directional voting method was utilized to calculate the frequency of positive correlation as well as the frequency of negative correlations between *GJA1* and each other gene. *Gja1* centric networks were then defined as a function of frequency threshold *n* (=1*, 2, …, 8*}.

### Construction of *GJA1* signaling map

Starting from *GJA1* as the root, we searched through the khaki module based Bayesian network for further three layers of genetic neighborhood, and trimmed away those single leaf nodes while keeping the remaining nodes. Then, we used the individual nodes or its first neighborhood in the remaining network to query the reactome (https://reactome.org/) and MSigDB database for gene ontology enrichment. A *GJA1* signal map was constructed by replacing each individual nodes in the network plot with a relevant gene ontology entry acquired from the query.

### Quantitative PCR

Quantitative PCR was performed as previously described [38]. Briefly, total RNA was isolated using Qiagen RNeasy kit, and 1–2 μg of total RNA was used to synthesize cDNA using Ecodry (Clontech, 639,543, Mountain view, CA). Universal probe library in combination with the primers listed (Additional file 1: Table S10) was used to perform qPCR with KAPA PROBE FAST qPCR master mix (KAPA Biosystems, KK4703, Wilmington, MA). Acquired Ct values were loaded onto qbase Plus software package (Biogazelle, Belgium) for data quality control and normalization. *Actb*, *Rpl13a*, and *Rplp0* were used as normalizer genes.

### Cell culture and reagents

Generation of *Gja1*−/− astrocytes were previously described [66]. WT and *Gja1*−/− primary astrocytes were prepared by dissecting and dissociating forebrains of P1–3 pups and culturing for 2 weeks in DMEM containing 10% fetal bovine serum and penicillin/streptomycin in T75 flask. Contaminating microglia were reduced by vigorous shaking flasks at 500 rpm for 20 min and replacement of medium containing floating cells. Mouse TNFα (14–8321-62) and IL-1β (14–8012-62) were purchased from eBiosciences and used at concentration of 10 ng/ml. Carbenoxolone (C4790), lanthanum (575275), quinine (Q1125) were purchased from Sigma-Aldrich (St. Louis, MO, USA).

### Neuron astrocyte cocultures

For RNA-seq in astrocytes and neuron/astrocyte cocultures, cortical neuron cultures were prepared from E15.5 wildtype C57BL6/J embryos as previously described [14]. 1 × 10^5^ neurons were seeded in poly-D-lysine coated 6 well plates with or without 1 × 10^5^ wildtype or *Gja1*−/− astrocytes in neurobasal medium (Life Technologies, 21,103–049, Grand Island, NY, USA) supplemented with B27 (Life Technologies, 17,504–044), and cultured for 10 days. At div10, half of medium was replaced with neurobasal containing 20 μM *A*β _1–42_ oligomer for four days, and total RNA was harvested using RNA-Bee (AMSBIO, Cambridge, MA, USA) following the manufacturer’s instruction. Four replicate wells were used for each condition.

For MEA assays, E15.5 cortical neuron cultures were prepared from wildtype C57BL6/J as above. MEA wells were coated with poly-D-lysine and one day prior to plating 1 × 10^5^ neurons, 5 × 10^4^ wildtype or *Gja1*−/− astrocytes were plated in neurobasal medium supplemented with B27. At days in vitro (div) 16, neuron/astrocyte cocultures were treated with 10 μM *A*β_1–42_ oligomers until div 20.

For neuronal death and viability assays, 2 × 10^5^ cortical neurons were plated onto PDL coated 12 well plate and 1 × 10^5^ wildtype or *Gja1−/*− astrocytes are plated in Transwells (Costar, 3460, Kennebunk, ME USA). At div7, Transwells with astrocytes were inserted to neuronal cultures and incubated with the indicated *A*β_1–42_ for 4 or 7 days. LDH assay was performed as in (Kajiwara, 2014) using LDH Cytotoxicity Detection Kit (Clontech, Mountain View, CA, USA). MTT assay was performed as described in (Bruban 2015).

### Apoe and Aβ ELISA

Astrocytes were seeded in 6 well plates at the density of 100,000 cells per well in serum free DMEM medium for 48 h with vehicle, 2 or 20 μM *A*β_1–42_ oligomers. Mouse Apoe secreted in conditioned medium was quantitated using mouse Apoe ELISA kit (Mybiosource, MBS705227, San Diego, CA, USA) following manufacture’s instruction. Astrocytes attached to the plate were used to prepare cell lysates as described below. Mouse amyloidβ 40 and 42 species were quantitatively determined using Human/Rat Amyloid ELISA kits (294–64,701 and 294–64,501, Wako Chemicals, Richmond, VA, USA).

### Immunoblotting

Lysates were prepared from astrocytes underwent above procedure in buffer containing phosphate buffered saline, protease inhibitor cocktail (Roche, Indianapolis, IN, USA), and 1x loading buffer. Lysates were resolved by denaturing SDS-PAGE gel and transferred to PVDF membrane. The membranes were blotted with following primary antibody: Cx43 (1:500, 3512, Cell Signaling Technology); Apoe (1:1000, sc-6384, Santa Cruz Biotechnology); Actin (1:1000, A5060, Sigma-Aldrich), and specific signals were detected with HRP-conjugated secondary antibodies followed by SuperSignal West Dura (34,075, ThermoFisher Scientific).

### Multielectrode array recordings

Recordings of neuronal activity was performed on Axion 768 channel system (The Maestro, Axion Biosystems, Atlanta, GA, USA) using 48-well plate, which records or stimulates neuronal activity from up to 16 electrodes per well. Manufacture’s recommended settings were used for detection of spikes, bursts and network bursts. Briefly, spikes were detected using Adaptive Threshold Crossing with 6x Standard Deviation; bursts were defined as minimum of 5 spikes with 100 ms of maximum inter-spike interval; and network bursts were defined as minimum of 10 spikes with 100 ms of maximum inter-spike interval and 20 ms of synchrony window. Spontaneous activities were recorded at days in vitro (div) 10, 14, 18, and 20 as follow. A MEA plate was set on heated stage at 37C for at least 5 min prior to initiation of recordings, which lasted for 10 min. All the recordings and analysis were performed on Axion Integrated Studio (Axion Biosystems). Stimulation was performed div 14 and 20 using the same spike and burst settings as in spontaneous recordings. A stimulation given was comprised of 3 repeated cycles of voltage-controlled (500 mV/− 500 mV), biphasic (500 μs each), and positive-first stimulus followed by 3 ms of floating duration [88], and was given to all electrodes. Stimulation was given 5 times with 5 s interval and stimulation data were analyzed using NeuralMetricTool (Axion Biosystems). Results are given as means of 5 stimulations.

### Statistics

All of the statistical significance levels reported in this study were corrected for multiple testing unless otherwise specified. Statistical analysis was performed using either R programming language or SPSS version 20 or higher (IBM corporation, Somers, NY, USA). Amyloid uptake assays were analyzed by Student’s t-test. Spontaneous neural recordings were analyzed by Man-Whitney, or Kruskal-Wallis non-parametric analysis.

## Results

**Fig. 1:**
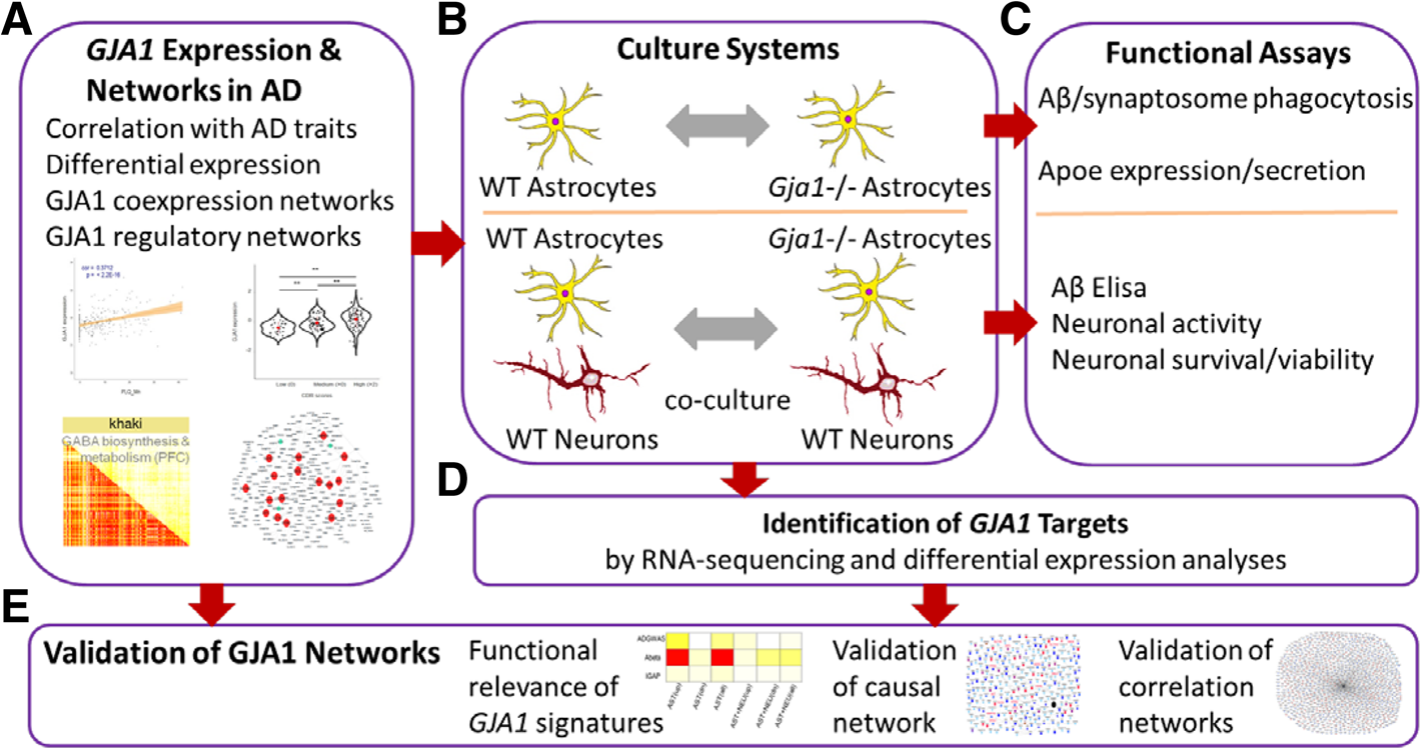
Overview of the integrative network analyses and validation experiments performed in the study. **a**. *GJA1* mRNA expression changes and its correlations with clinical and pathological traits were systematically investigated in 29 datasets. *GJA1*-centric coexpression and regulatory networks. **b**. Workflow of in vitro functional validation study. Wildtype and *Gja1*−/− astrocytes with or without wildtype neurons were used to prepare RNA for sequencing and to perform various functional validations (**c**). **d**. *GJA1*’s gene signatures between wildtype and *Gja1*−/− astrocytes and between coculture of wildtype astrocytes and wildtype neurons and coculture of *Gja1*−/− astrocytes and wildtype neurons were identified from RNA-sequencing data from the experiments in **b**. **e**. *GJA1*’s gene signatures were used to validate the network structures predicted from the transcriptomic datasets in human AD brains. Functional relevance of *GJA1*’s gene signatures was also investigated

Figure 1 shows the workflow of the integrative network analysis and functional validation experiments performed in this study. We first examined the relationships between *GJA1* mRNA expression and clinical and pathological traits in 29 AD transcriptomic datasets. We also investigated the role of *GJA1* in gene networks underlying AD. We then performed in vitro experiments to study the role of *GJA1* in regulating AD gene networks and AD related phenotypes using primary astrocytes purified and cultured from wildtype and astrocyte specific *Gja1*−/− mice. *Gja1*’s target gene signatures identified from the RNA-seq data from the in vitro experiments were then projected onto the *GJA1* centric networks to validate these networks’ structures.

### *GJA1* is a key regulator of an astrocyte specific gene subnetwork dysregulated in LOAD

Several studies have used co-expression network analysis to find modules of co-regulated genes in AD [36, 60, 61]. Our previous study developed a novel network approach capable of integrating clinical and neuropathological data with large-scale genetic and gene expression [98]. This network biology approach led to a novel multiscale network model of LOAD, which identified a number of coexpressed gene modules that were strongly associated with AD pathological traits or underwent dramatic disruption of high-order gene-gene interactions [98]. One such module, referred to as the khaki module in the original construction of this network, was of particular interest since it included *APOE*, the top AD risk factor gene. Moreover, the average interaction strength among its member genes in LOAD was reduced by 71% compared to that in normal control at a false discovery rate (FDR) < 2%, suggesting a huge loss of coordination among this group of genes in AD. The khaki module was enriched for the genes in Gamma-aminobutyrate (GABA) biosynthesis and metabolism (24 fold enrichment (FE), Fisher’s exact test (FET) *p* = 0.046) and harbored 12 (*ALDOC, APOE, AQP4, ATP1A2, CSPG3, CST3, EDG1, EMX2, GJA1, PPAP2B, PRDX6* and *SPARCL1*) of 46 known astrocyte marker genes, a 15-fold enrichment over what would be expected by chance (FET *p* = 6.55E-9). The module was also enriched for the expression of the common variants identified as genome-wide significant by AD genome wide association studies (GWAS) (3-FE, FET *p* = 1.92E-11). Bayesian causal network analysis showed that *GJA1* was the top driver of the module followed by *FXYD1*, *STON2* and *CST3* [98]. The key drivers of the corresponding causal network of the module were the nodes that had a large number of downstream nodes [90, 98]. These results indicate that *GJA1* is a potential regulator of molecular networks in AD. In the next subsection, we will investigate the association between *Gja1* mRNA expression and AD. All of the statistical significance levels reported were corrected for multiple testing unless otherwise specified.

### *GJA1* expression is associated with AD clinical and pathophysiological traits

To gain insight into the role of *GJA1* in cognitive functions and AD pathogenesis, we first extensively investigated how *GJA1* expression at the mRNA level was correlated with AD neuropathological traits in 29 gene expression datasets from three AD cohort studies of aging and dementia that included organ donation at death: the Mount Sinai/JJ Peters VA Medical Center Brain Bank (MSBB; Additional file 1: Table S1) [91], the Religious Orders Study and the Rush Memory and Aging Project (ROSMAP) [8, 9] and in the Harvard Brain Tissue Resource Center Alzheimer’s Disease study (HBTRC) [98]. We chose six different clinical and pathological criteria to evaluate the clinical relevance of *GJA1* on AD pathology and cognitive functions: the MiniMental State Examination (MMSE) score [25, 32], the sum of NFT density estimates for all cortical regions examined (NTrSum), Mean Plaque density (PLQ_Mn) for the estimation of average plaque density, Braak stage score for quantitative assessment of neurofibrillary tangles [11], the Consortium to Establish a Registry for Alzheimer’s disease (CERAD: 1 for definite AD, 2 for probable AD, 3 possible AD, 4 for normal control) score for quantitative measure of neuritic plaques, and clinical dementia rating score (CDR ranging between 0 and 5 with 0 for normal control and 5 for severe dementia).

**Fig. 2:**
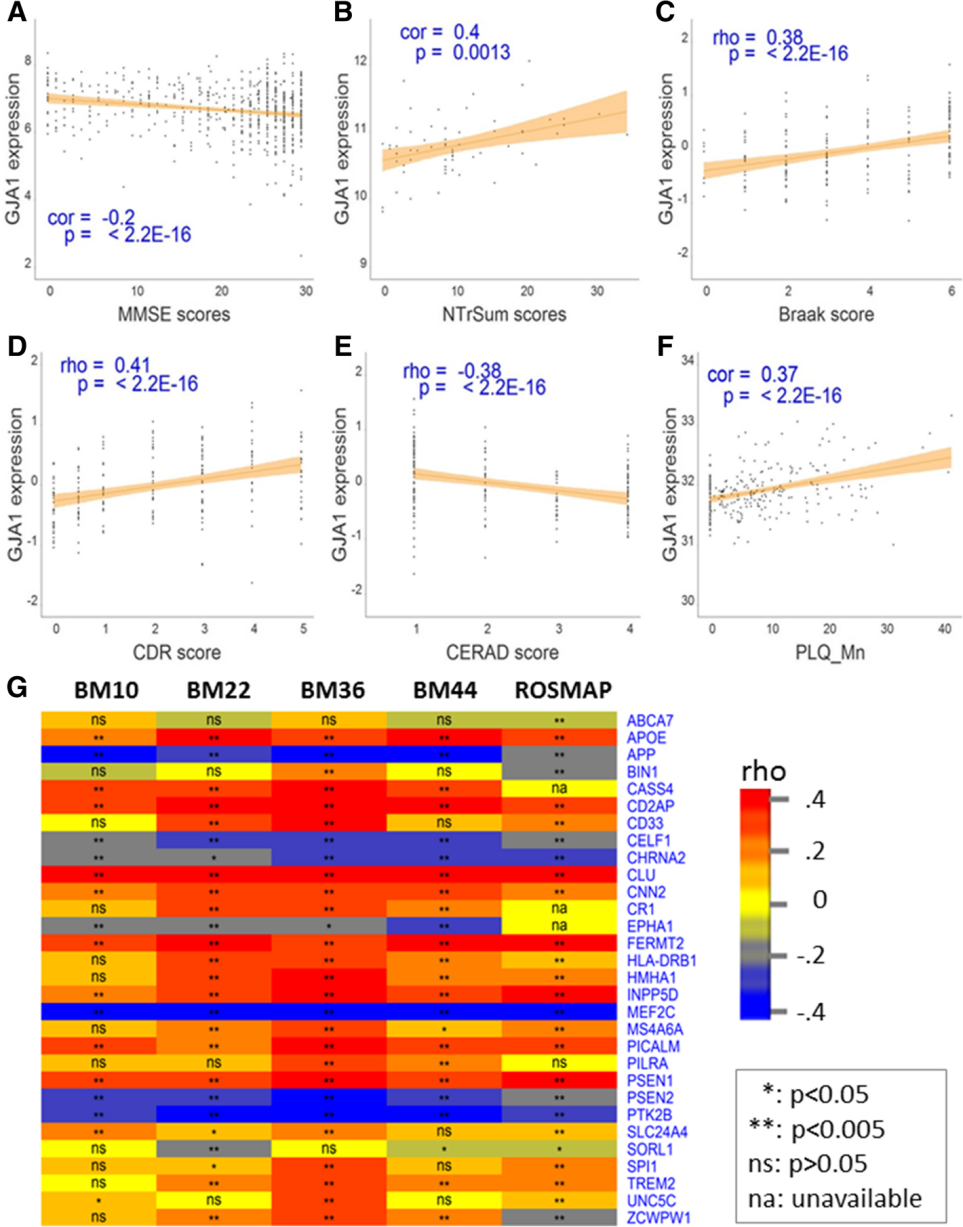
*GJA1* mRNA/protein expression is correlated with AD pathogenesis, dementia and known AD risk factor genes. *GJA1* mRNA expression is correlated with the MMSE score in the prefrontal cortex region in the ROSMAP cohort (**a**), the NTrSum score in the cortex region frontal pole (BM10) the MSBB cohort (**b**), the Braak score in the parahippocampal gyrus (BM36) in the MSBB cohort (**c**), and the CDR score in BM36 in the MSBB cohort (**d**), and CERAD in BM36 in the MSBB cohort (**e**). The GJA1 protein level is also correlated with the plaque mean density (PLQ_Mn) in the frontal pole (BM10) in the MSBB cohort (**f**). Inset was the correlation coefficient along its *p* value between individual clinical traits and *GJA1* mRNA/protein level. Severity of AD symptoms was classified based on each individual AD neuropathological and cognitive traits according to the criteria established in the ROSMAP clinic codebook [91, 92]. **g**. *Gja1* mRNA expression is correlated with a vast majority of the ADGWAS genes in the MSBB (BM10, BM22, BM36 and BM44) and ROSMAP RNA-seq datasets. Colors represent Correlation coefficients: red, high; blue, low; yellow, in between. * and ** stand for significance at 5 and 0.5% level, respectively while ns indicates insignificant correlation and na stands for “not applicable”. Shown in the heatmap are only the ADGWAS genes that passed RNA-seq data preprocessing, normalization and annotation

In the microarray data in the ROSMAP cohort, *GJA1* expression was significantly correlated with CERAD score (*r* = − 0.15, *p* = 3.3E-3) and the MiniMental State Examination (MMSE) score (*r* = − 0.14, *p* = 6.4E-3). Similar results were observed in the ROSMAP RNA-seq dataset (Fig. 2a, and Additional file 1: Table S2), suggesting that the mRNA expression of *GJA1* is associated with AD pathogenesis and dementia. The MSBB AD cohort includes microarray and RNA-seq data from a battery of distinct brain cortical regions and thus provides an excellent opportunity to investigate regional differences in the correlation between *GJA1* expression and AD neuropathological traits [91]. Among the 19 brain cortex regions investigated in the MSBB AD microarray data, *GJA1* expression in six cortex regions including BM10 (frontal pole), BM20 (inferior temporal gyrus), BM21 (middle temporal gyrus), BM32 (anterior cingulate), BM36 (parahippocampal gyrus) and BM46 (dorsolateral prefrontal cortex) was significantly correlated with at least three AD neuropathological traits (Fig. 2b, and Additional file 1: Table S2). Overall, *GJA1* expression in these six cortex regions displayed a significant positive correlation with Braak stage score, PLQ_Mn, NTrSum and CDR. The MSBB AD RNA-seq data revealed a consistent pattern of correlation between *GJA1* expression and AD clinic traits across the cortical regions studied **(**Fig. 2c-e; Additional file 1: Table S2). Notably, in BM10, BM36 and BM44 cortex regions, the microarray and RNA-seq data converged to show a consistent correlation between *GJA1* expression and AD neuropathological traits (Additional file 1: Table S2). Thus, in the MSBB cohort, the association between *GJA1* expression and AD neuropathological traits was cortex-specific. At the protein level, GJA1 in the brain cortex BM10 region was significantly correlated with CERAD (*r* = − 0.32, *p* = 1.26E-07), PLQ_Mn (*r* = 0.37, *p* = 4.08E-10) (Fig. 2f) and CDR (*r* = 0.35, *p* = 5.69E-9) (Additional file 1: Table S2). Also the total soluble amyloidβ (Aβ) levels had a significant positive correlation with GJA1 protein levels in the BM10 region (*r* = 0.18, *p* = 0.0036).

We showed previously in the HBTRC cohort that *Gja1* had a significant correlation with Braak score (*r* = 0.61 and 0.52 for dorsolateral prefrontal cortex and cerebellum cortex regions, respectively) [98].

We further checked how *GJA1* mRNA expression was correlated with 30 known AD risk factor genes. As shown in Fig. 2g, a majority of those AD genes were significantly correlated to *GJA1* in the five RNA-seq datasets.

We also examined *GJA1* differential expression between various subgroups of AD severity with respect to each individual AD neuropathological or functional cognitive trait using pairwise Student’s t-test. Consistent with the correlation analysis, *Gja1* expression increased significantly as the disease deteriorated (Additional file 1: Table S3).

Because *APOE* is one of the major AD risk factors, and age and sex are critical clinic covariables in AD neuropathology, we investigated whether age, sex and APOE genotypes had any impact on the association of *GJA1* expression with AD clinical and pathological traits. We stratified this cohort by age of death (AOD) (AOD > 85 versus AOD < 85), sex (female versus male), and *APOE* genotypes (E23, E34 and E33). As demonstrated in Additional file 1: Table S4 and Additional file 2: Figure S1, *GJA1* expression is more significantly associated with clinical dementia rating (CDR) in the group with AOD > 85 than that with AOD < 85, in females than males, and in the group with *APOE* E33 than that with E34 or E23, across four brain regions in the MSBB cohort, suggesting that age, sex and *APOE* genotypes impact the association of *GJA1* expression with clinical and pathological traits.

We further assessed if *GJA1* mRNA expression was correlated with the variants of the known AD risk genes using the RNA-seq data from the brain region BM36 in the MSBB cohort. Among the 28 ADGWAS genes that had identifiable variants in the present study, 18 had at least one variant that possessed a significant correlation with *GJA1* (Additional file 2: Figure S2). For example, the transcript/isoform ENST00000532146 of the risk factor *CELF1* is significantly correlated with *GJA1* expression in BM10 and BM44, but not BM22 and BM36 while ENST00000534614 and ENST00000539254 are correlated with *GJA1* expression only in BM36 and BM22, respectively (Additional file 2: Figure S2). These results suggested that variation in transcripts’ abundance might be an important factor for determining the co-regulation between *GJA1* and AD risk factors.

In summary, both correlation and differential expression analyses revealed that *GJA1* was associated with amyloid and tau pathologies of AD as well as cognitive functions suggesting that *GJA1* may play an important role in AD.

### Transcriptomic changes caused by *Gja1* deficiency in mouse astrocytes

**Fig. 3:**
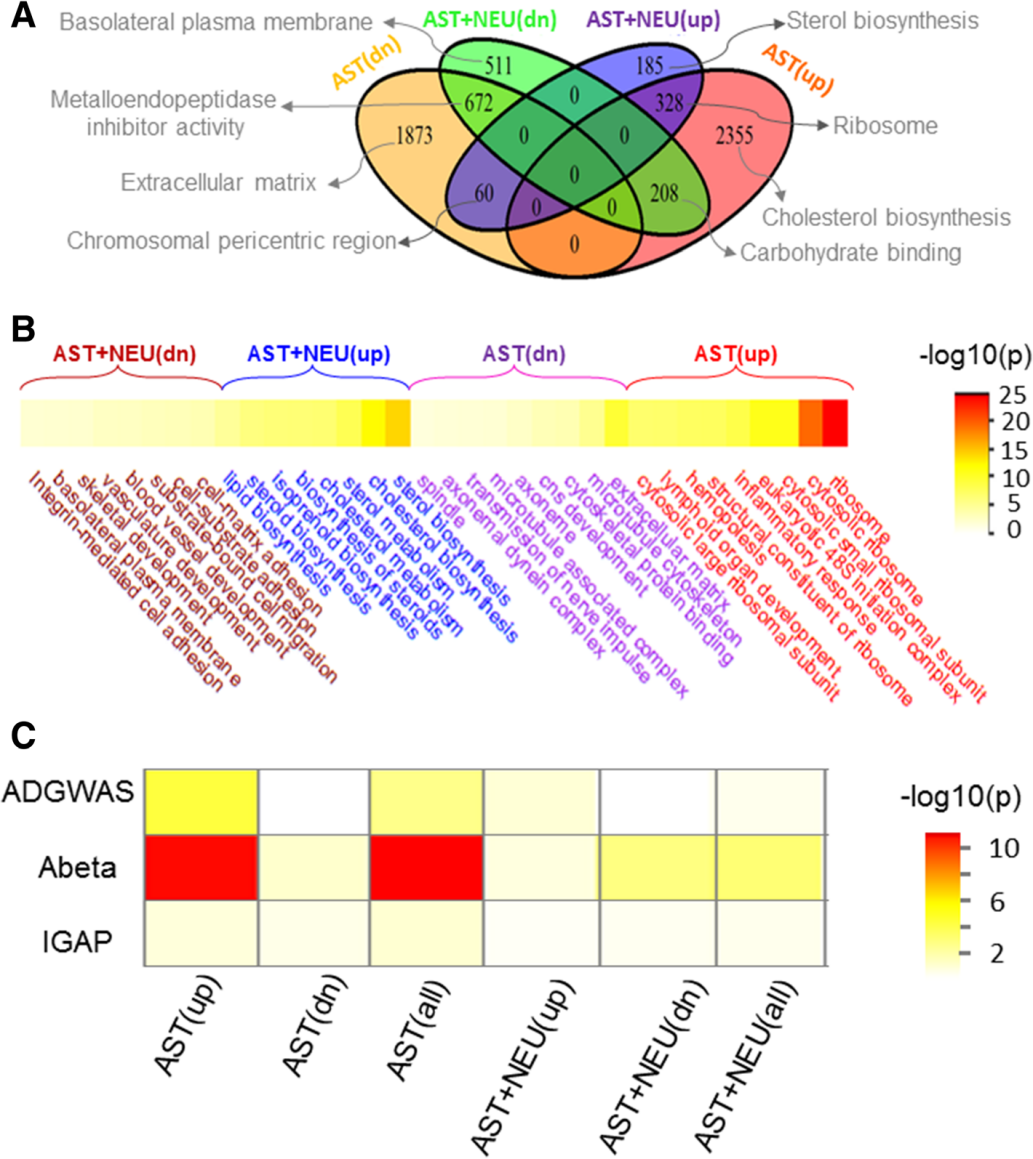
The impact of GJA1 deficiency on AD relevant gene ontologies and AD risk networks. **a.** Venn diagram for genes overlapping among various differentially expressed genes (DEG) signatures. AST(dn) and AST(up) were down- and up- regulated DEG signatures in Gja1−/− vs. wildtype astrocyte cultures, while AST + NEU(dn) and AST + NEU(up) were down- and up- regulated DEG signatures in Gja1−/− vs. wildtype astrocytes co-cultured with neurons. Top gene ontology for each individual DEGs were shown above each cluster. **b**. Heatmap of Gene enrichment significance (−log10(p)) for the DEG signatures. Top 10 enriched gene ontologies were shown for each individual DEGs. **c**. *GJA1*−/− signatures including AST(all), AST(up), AST(dn), AST + NEU(all), AST + NEU(up) and AST + NEU(dn) are enriched for three AD genetic gene sets including the one identified by the International Genomics of Alzheimer’s Project (IGAP) [1], the Abeta (Aβ) genetic network [16], the AD risk gene list (ADGWAS) [43]

To validate the role for *GJA1* in orchestrating the astrocytic transcriptome, we purified and cultured primary astrocytes from wildtype and astrocyte specific *Gja1*−/− mice, and identified differentially expressed genes (DEGs) by RNA-seq in *Gja1*−/− vs wildtype primary astrocytic cultures in the absence or presence of wildtype primary cortical neurons. Each group had four replicates. All cultures were treated with 10 μM *A*β_1–42_ oligomer from div 10 through 14, when total RNAs were harvested. We identified 2891 upregulated (termed AST(up)) and 2605 downregulated (termed AST(dn)) DEGs upon the ablation of the *Gja1* gene as compared to wildtype primary astrocytes (Fig. 3a). We identified 573 upregulated genes (termed AST + NEU(up)) and 1391 downregulated genes (termed AST + NEU(dn)) in *Gja1*−/− vs. wildtype primary astrocytes co-cultured with primary neurons (Fig. 3a). AST(up) shared 328 genes (11.3%) with AST + NEU(up) and 208 genes with AST + NEU(dn) while AST(dn) shared 672 and 60 genes with AST-NEU(dn) and AST-NEU(up), respectively (Fig. 3a). These DEG signatures were enriched for a variety of biological pathways including translational processes, immune response, cell-cell communication, extracellular matrix, microtubule cytoskeleton, synaptic transmission, lipid biosynthesis, steroid biosynthesis, cholesterol biosynthesis and cell-matrix adhesion (Fig. 3a-b, Additional file 1: Table S5).

AST(up) was significantly enriched for the genes in an Aβ network signature [16] (2.03 FE, FET *p* = 1.08 E-10), the AD genome-wide significant risk factor gene signature (ADGWAS) (3.47 FE, FET *p* = 6.58E-6) (Fig. 2c) while AST + NEU(dn) was enriched for the genes in the Aβ network signature (1.68 FE, FET *p* = 1.1E-3) (Fig. 3c and Additional file 1: Table S6). These results suggested that *Gja1* is a critical regulator of the AD GWAS genes and may play an important role in Aβ metabolism.

We further intersected the *GJA1* KO DEG signatures with the signatures from other inflammatory diseases to better understand of the immune response component in *GJA1* regulated gene expression. We considered 2 well-established inflammatory gene signatures, an inflammatome signature of 2461 genes from eleven rodent inflammatory disease models [90] and the human macrophage and immune response enriched module of 2483 genes causally linked to obesity and diabetes [23]. These two inflammatory signatures share 758 genes [termed the core disease-related inflammatory gene (CDIG)]. About half of the CDIGs fall into AST(up) (accounting for about 12.6% of AST(up), 6.57 FE, FET *p* = 1.85E-208) and the intersection includes well known inflammatory markers (e.g., *CD44, CD53, FCER1G, HCK, TYROBP* and *TREM2*), inflammation complement component members (*C1QA, C1QB,* and *C1QC*), CXC chemokines (*CXCL10, CXCL3* and *CXCL6*) and TNF Receptors (*TNFRSF11B* and *TNFRSF13B*). The CDIGs are also significantly enriched in AST + NEU(up) (accounting for about 15% of AST + NEU(up), 7.84 FE, FET *p* = 1.06E-49). These results suggest that inflammation is a critical component in *GJA1*-regulated gene expression.

We then investigated expression changes of astrocyte- and neuron-specific genes in various gene signatures using the recently identified brain cell type specific signatures [57]. Astrocyte- and neuron-specific marker gene signatures are enriched in the down- and up-regulated gene signatures in the neuron and *Gja1*−/− astrocyte co-cultures versus the neuron and wildtype astrocyte co-cultures, respectively (Additional file 1: Table S9A). The co-culture systems with and without *Gja1*−/− upregulated a significant portion of the neuron-specific marker genes when compared with the respective astrocyte alone models (with or without *Gja1−/−*) while down-regulating many astrocyte marker genes (Additional file 1: Table S9B). The gene lists from the abovementioned intersection analyses can be found in (Additional file 1: Tables S9C and S9D).

### *Gja1* deficiency induced transcriptomic changes highly overlap the *GJA1* centric gene networks in AD

We further examined the molecular mechanisms of *GJA1* in AD pathogenesis and cognitive function. We first examined if these *Gja1−/−* DEG signatures were enriched in the Khaki module where *GJA1* resided based on our previous study [98]. As shown in Additional file 1: Table S7, AST + NEU(dn), AST(dn) and AST(up) were all significantly enriched in the khaki module with FET *p* = 3.74E-54 (9.0 FE), 3.64E-28 (4.4 FE), 1.28E-5 (2.1 FE), respectively. On the other hand, the overall signatures AST(all) and AST + NEU(all) also significantly overlapped the module with FET *p* = 3.03E-32 (3.2FE) and 1.9E-47 (6.7 FE), respectively .

**Fig. 4:**
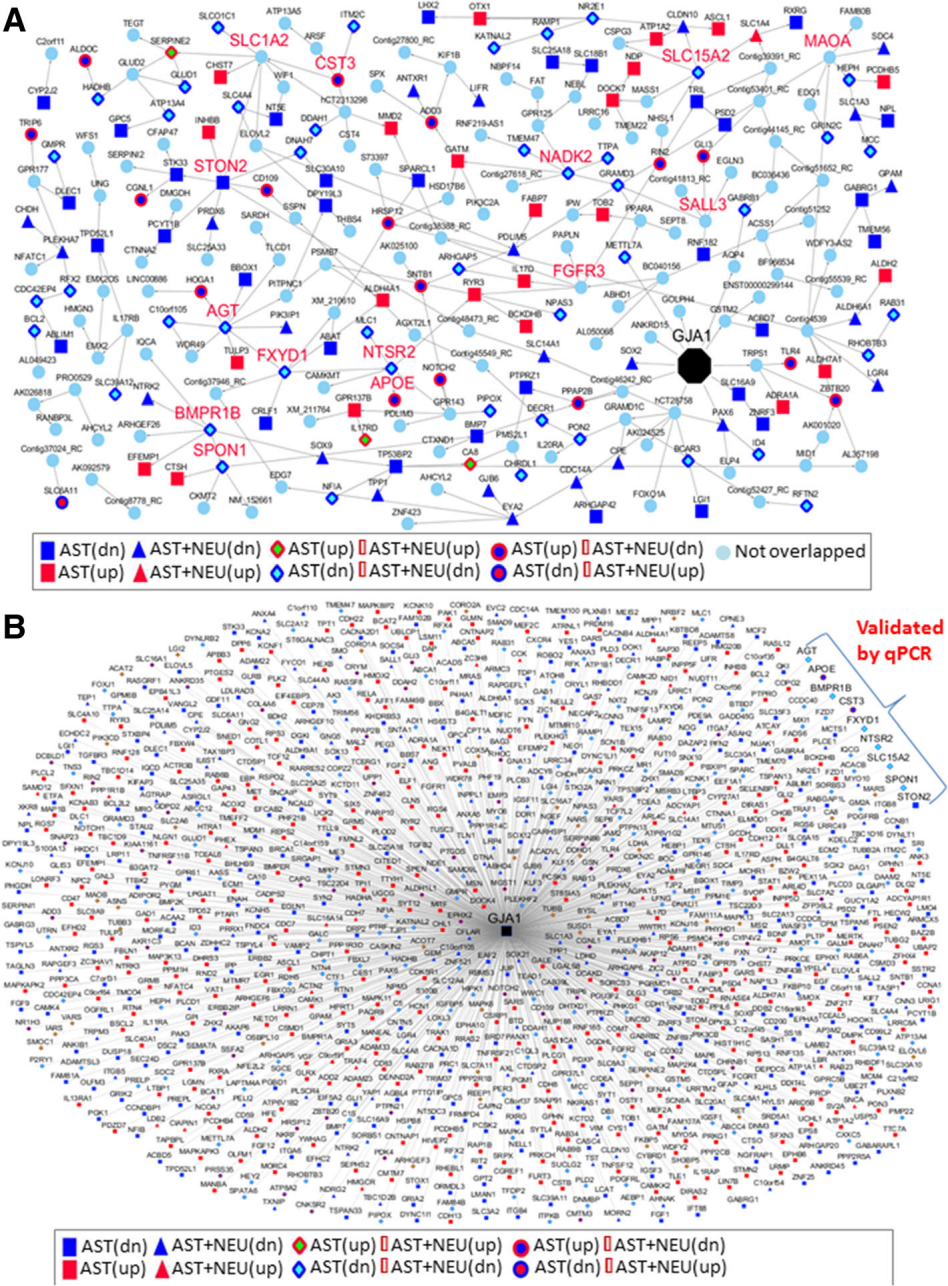
Network analysis on GJA1 genetic networks. **a**. BN (Bayesian Network) based on astrocyte (khiki) module. *Gja1−/−* DEG signatures were projected onto BN to detect overlapped gene signatures, which were classified into eight categories: up-regulated only in AST (in red upright triangle), down-regulated only in AST (in blue downside triangle), only up-regulated in AST + NEU (red square), only down-regulated in AST + NEU (blue square), up-regulated in both AST and AST + NEU (in pink diamond), up-regulated in AST but down-regulated in AST + NEU (in pink hexagon), down-regulated in both AST and AST + NEU (in gray diamond), and down-regulated in AST but up-regulated in AST + NEU (in gray hexagon)). In enlarged label were genes that were validated experimentally. *Gja1* was in black. In turquoise circle were genes non-overlapped. AST, *Gja1−/−* astrocyte culture while AST + NEU, *Gja1−/−* astrocyte culture in the presence of co-cultured neurons. **b**. GJA1 centered genetic networks inferred by projecting *Gja1−/−* DEG signatures onto Gja1 correlation concensus network (CGCCS(6)). Overlapped gene nodes were denoted as in A

To gain more insights into the signaling circuit of the *GJA1* regulation in AD, we constructed the Bayesian causal network for the khaki module and projected the *Gja1−/−* DEG signatures onto this network in order to delineate the underlying causal relationship among the molecular constituents of this module. As shown in Fig. 4a and Additional file 1: Table S7, 13 of the 19 predicted key drivers except for *GJA1* in the khaki module were regulated by *GJA1* (4.94 FE, FET *p* = 4.70E-05). Strikingly, 11 out of the 13 *GJA1* regulated key driver genes were within the *GJA1’s* downstream network neighborhood (Additional file 2: Figure S3A). To better gain insights into the functions of the Bayesian causal network in Fig. 4a, we built up a *GIA1* signaling pathway map. As shown in Additional file 2: Figure S3B, *GIA1* regulates a diversity of pathways such as regulation of gap junction activity, innate immune system, TGFβ signaling, DNA repair and lipid metabolism. These results suggested that *Gja1* deficiency significantly impacted the khaki module and thus strongly validated our previous prediction of *GJA1* as a driver of this important AD related subnetwork.

To more comprehensively determine *GJA1*’s target genes in AD, we further systematically identified the genes correlated with *GJA1* in a number of human AD cohorts. Genes significantly correlated with *GJA1* (Benjamini-Hochberg (BH)-corrected *p*-value < 0.05) were first identified in each of eight datasets including four from the MSBB RNA-seq cohort, one from the ROSMAP RNA-seq data and three from the HBTRC cohort. Note that only the data from the AD subjects were used for the correlation analysis. A majority voting method was utilized to calculate the frequency of each correlation across the eight datasets (Additional file 1: Table S8). We defined consensus *GJA1*-centered correlation signature (CGCCS) as a function of frequency threshold *n*, i.e., CGCCS(*n*) = {g | frequency(r(*g*, *GJA1*)) ≥ *n*}, where r(g, *GJA1*) represents a significant correlation between a gene *g* and *GJA1* at FDR < 0.05, and *n* = 1, 2, …, 8. This process led to *GJA1* centered correlation networks, also denoted as CGCCS(n).

Additional file 2: Figure S4 shows the enrichment of the *GJA1* centered correlation networks for the previously identified DEGs in AST and AST + NEU. CGCCS (4) was most significantly enriched for the AST(all) and AST + NEU(all) DEG signatures with FET *p* = 1.0E-330 (3.2 FE) and 7.5E-311 (3.9 FE), respectively (Black lines, Additional file 2: Figure S4). CGCCS (6) is enriched for the DEG signature in AST with FET *p* = 6.2E-309 (3.8 FE). Figure 4b shows the network CGCCS (6) which includes 201 up-regulated (red nodes, termed GJA1_centered_AST(up)) and 307 down-regulated (blue nodes, termed GJA1_centered_AST(dn)) genes upon the knockout of *Gja1* in astrocytes. About 30% of the genes in each of the two *GJA1_centered_*signatures belonged to the khaki module (FET *p* = 1.52E-90, 32.37 FE). This *GJA1*-centered correlation network included eight driver genes (*AGT, BMPR1B, CST3, FXYD1, NTSR2, SLC15A2, SPON1, and STON2)* in the khaki module [98]. Similar results were derived for the AST + NEU(up) and AST + NEU(down) signatures. Therefore, *GJA1* impacts the expression of many key network drivers in the astrocytic subnetwork, suggesting its central role in this AD-related gene network. Furthermore, the analysis of the genes specific to the A1/A2 astrocytic activation in our DEG from *Gja1*−/− astrocytes [50] revealed that most of pan-, A1-, and A2-specific genes were generally upregulated (Additional file 2: Figure S5).

### Inflammatory cytokines downregulate expression of *Gja1* and the astrocytic subnetwork module

**Fig. 5:**
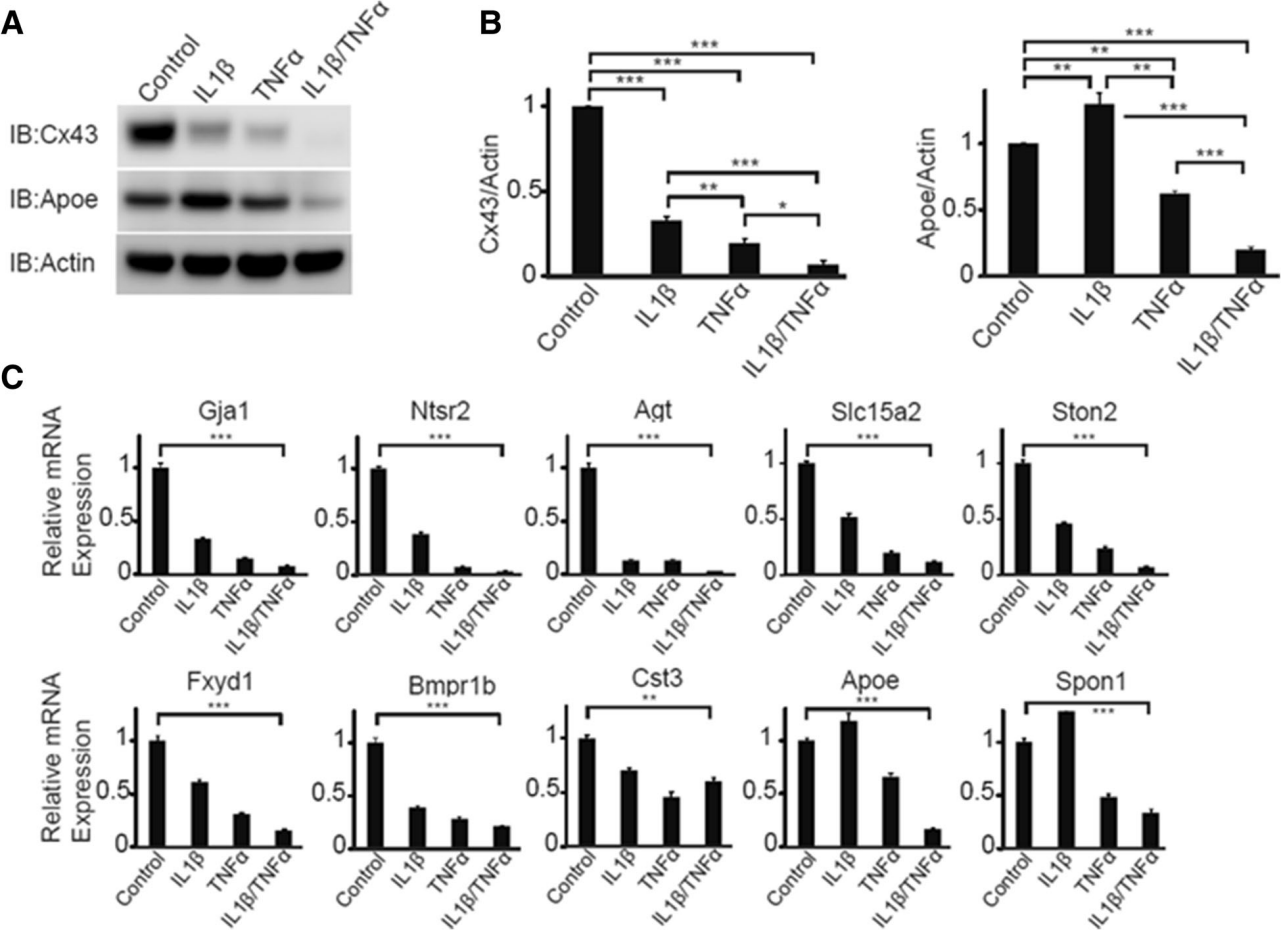
Inflammatory cytokines downregulates Gja1, Apoe, and other network genes. **a**. Wildtype primary mouse astrocytes were treated with either IL-1β (10 ng/ml) or TNFα (10 ng/ml) or in combination for 7 days and levels of Cx43, Apoe expression were analyzed by immunoblot. Representative results from 4 (*Gja1*) and 3 (Apoe) independent experiments are shown. **b**. Protein levels of Cx43 (left) and Apoe (right) were quantitatively analyzed for these technical replicates after normalization to Actin. ANOVA followed by Bonferroni post-hoc tests are indicated by asterisks. * *p* < 0.05, ** *p* < 0.01, *** *p* < 0.001. **c**. Quantitative gene expression analysis in wildtype astrocytes with treatments similar to **a**) was performed to analyze the other key drivers of the *GJA1*-centered network. Results representative of two independent experiments are shown. Statistical analysis was performed as in B, and only the comparison between control and IL-1β/TNFα is shown

Previously it has been shown that LOAD-relevant inflammatory cytokines such as TNFα and IL-1β downregulated expression of *Gja1* in astrocytes [19, 74]. Wildtype astrocytes were treated with TNFα, or IL-1β, or both for 7 days, confirming that Cx43 (Gja1 protein) was profoundly and synergistically reduced by both cytokines (Fig. 5a-b). Paradoxically, IL-1β significantly increased, but TNFα significantly decreased, Apoe protein levels (Fig. 5b). Following cytokine treatment, we tested *Gja1*, *Apoe* and eight key network driver genes found to be differentially expressed in *Gja1−/−* astrocytes by RNA-seq analysis, as a proxy to capture the network changes. qPCR analysis revealed that *Gja1, Apoe* and the other astrocytic subnetwork drivers were similarly downregulated by these cytokines (Fig. 5c).

### Gja1 channel activity increases the expression of the astrocytic subnetwork module

**Fig. 6:**
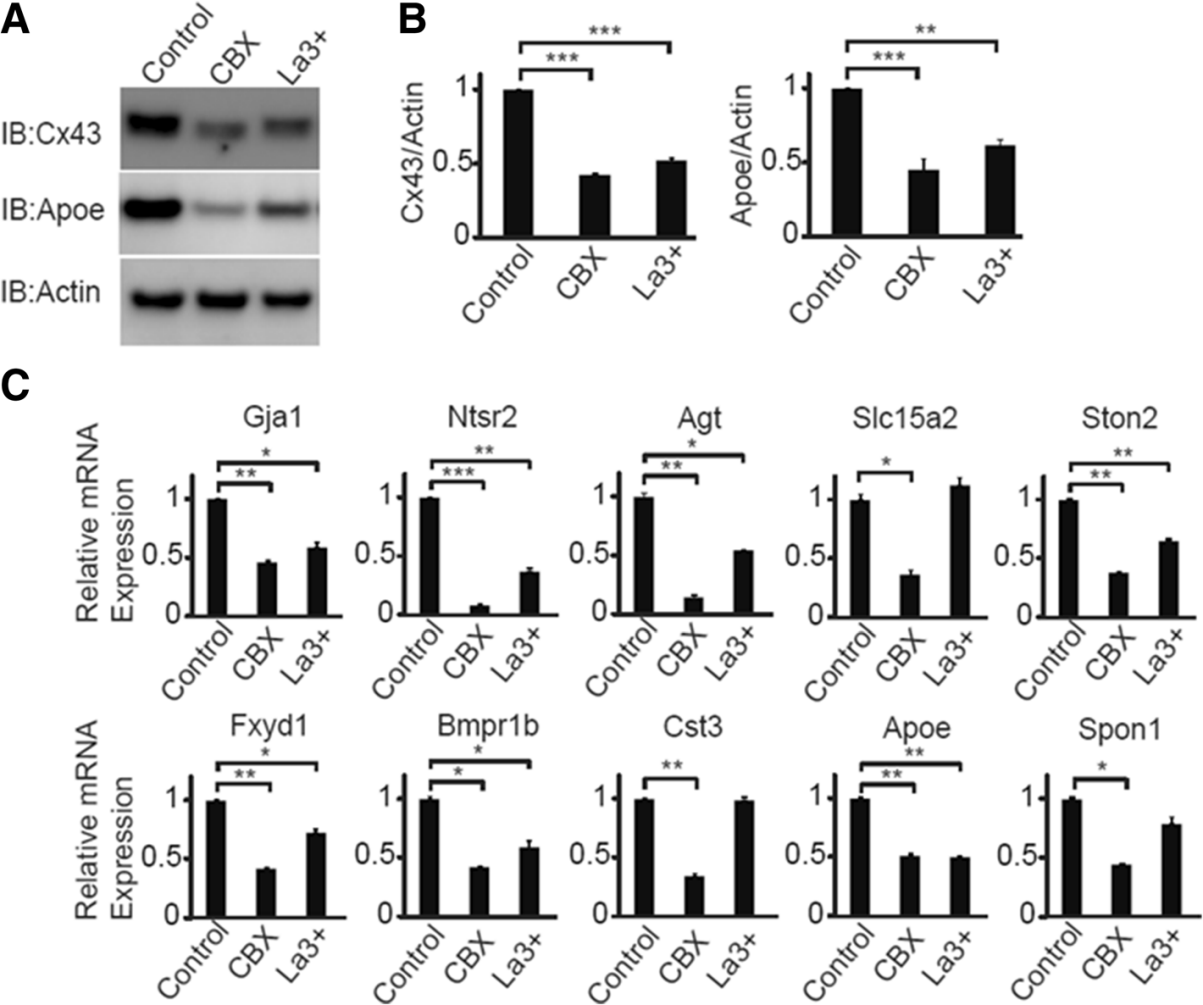
*GJA1* inhibitors downregulate *Gja1* and Apoe, and other network genes. **a**. Wildtype primary mouse astrocytes were treated with 200 μM carbenoxolone (CBX, gap junction inhibitor) or Lanthanum (La3+, hemichannel inhibitor) for 3 days and levels of Cx43, Apoe were analyzed by immunoblot. **b**. Quantitative analysis of Cx43 and Apoe protein levels were normalized to Actin. ANOVA followed by Bonferroni post-hoc tests are indicated by asterisks. ** *p* < 0.01, *** *p* < 0.001. **c**. Quantitative gene expression analysis in wildtype astrocytes treated similar to A was performed to analyze GJA1 network drivers. Statistical analysis was performed as in **b**, and only the comparisons to control are shown. Results representative of two independent experiments are shown for all experiments

Since these cytokines inhibit GJC and potentiate hemichannel activities [74], we asked whether inhibition of GJC and hemichannel activities regulates *Gja1* and other astrocytic subnetwork drivers. Treatment of wildtype astrocytes with carbenoxolone (CBX, inhibitor of GJC and hemichannel [2, 95]) or lanthanum (La3+, inhibitor of hemichannel [2]) led to significant reduction of *Gja1* and Apoe protein levels (Fig. 6a-b). Interestingly, the CBX treatment had broader effects on the reduction of the driver genes (Fig. 6c), while La3+ had generally milder and more selective effects, suggesting that GJC and hemichannel activities contribute to the regulation of distinct sets of the genes.

We next tested whether *Gja1* channel activation can alter the astrocytic subnetwork. *Gja1* hemichannel activity can be increased by quinine [81]. Treatment of wildtype astrocytes with quinine significantly upregulated Gja1 and Apoe protein and mRNA levels, along with transcriptional upregulation of a subset of the driver genes (Additional file 2: Figure S6). These data collectively supports *Gja1*, and specifically its channel activity, as an important regulator of astrocytic gene coexpression network including *Apoe*.

### Increased neuronal survival following Aβ treatment when co-cultured with *Gja1−/−* astrocytes

**Fig. 7:**
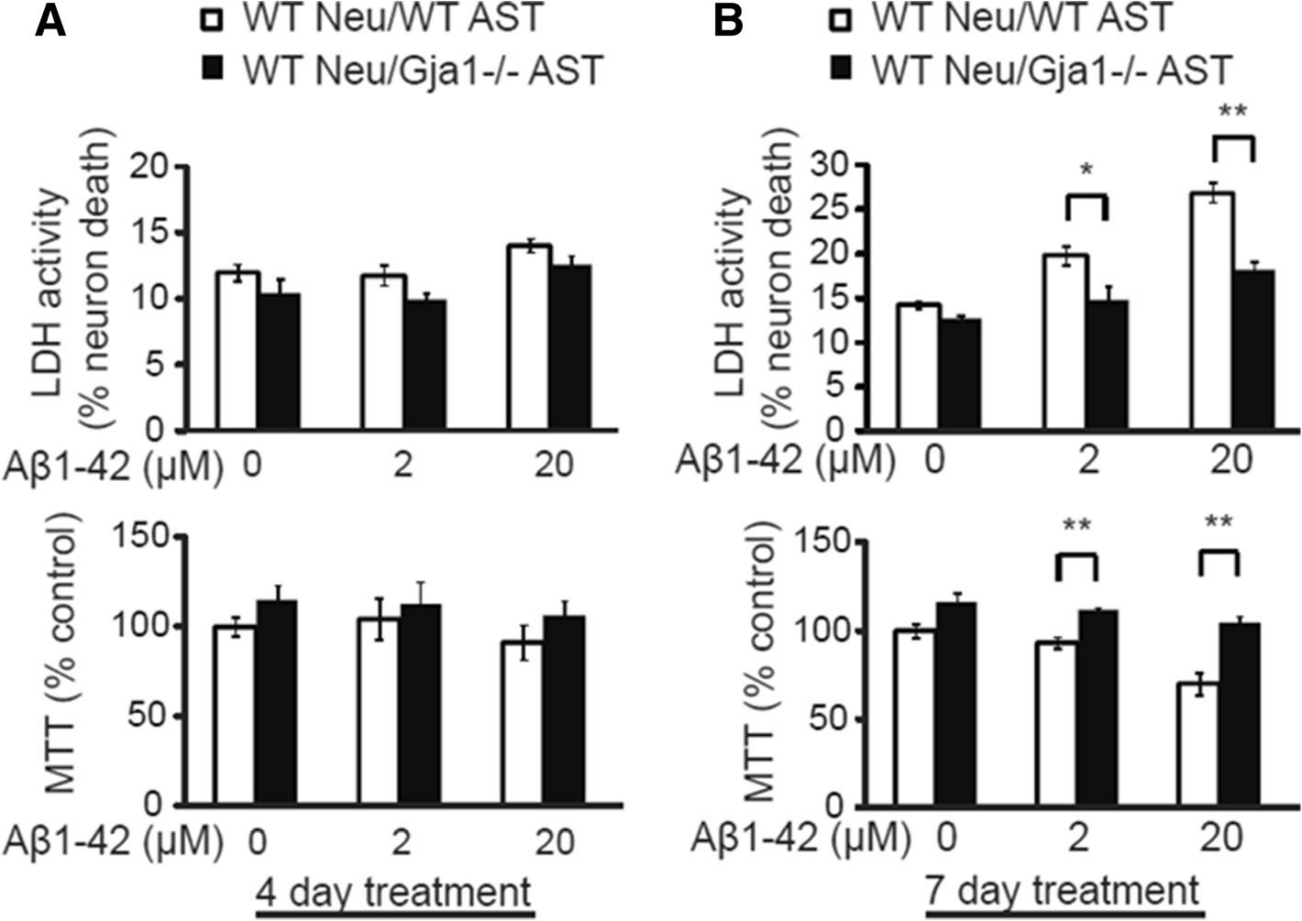
*Gja1* contributes to neuronal death and reduces viability by *A*β_1–42_ treatment. Cortical neurons were plated onto 12 well plate and wildtype or *Gja1*−/− astrocytes grown on transwells were inserted to the neuron cultures. Indicated concentration of *A*β_1–42_ oligomers are applied for 4 days (**a**) or 7 days (**b**) and neuronal death and viability was determined by LDH and MTT assays, respectively. Student’s t-tests were used for all the statistical analysis. * *p* < 0.05, ** *p* < 0.01, *** *p* < 0.001. For all experiments, representative of at least two independent experiments is shown

We assessed the role for *Gja1* in neuronal death and viability in a transwell co-culture system, in which cortical neurons were grown on the bottom of the plate, while astrocytes were cultured on the transwell insert. This allowed physical separation of the two cell types, while maintaining chemical continuity. Incubation of wildtype neurons and wildtype astrocytes with the indicated concentration of *A*β_1–42_ oligomers resulted in increased neuronal death [t (4) = − 2.941, *p* = 0.030 at 2 μM; t (4) = − 5.857, *p* = 0.004 at 20 μM] and decreased viability [t (6) = − 5.395, *p* = 0.002 at 2 μM; t (6) = 4.671, *p* = 0.003 at 20 μM] at day 7 (but not at day 4 (Fig. 7a)), in a dose dependent manner (Fig. 7b). Wildtype neurons cultured with *Gja1−/−* astrocytes were significantly resistant to death and loss of viability (Fig. 7b), consistent with a previous study showing that *Gja1−/−* deficient astrocytes conferred neuroprotection [69].

### Reduced Apoe secretion and synthesis in *Gja1−/−* astrocytes

**Fig. 8:**
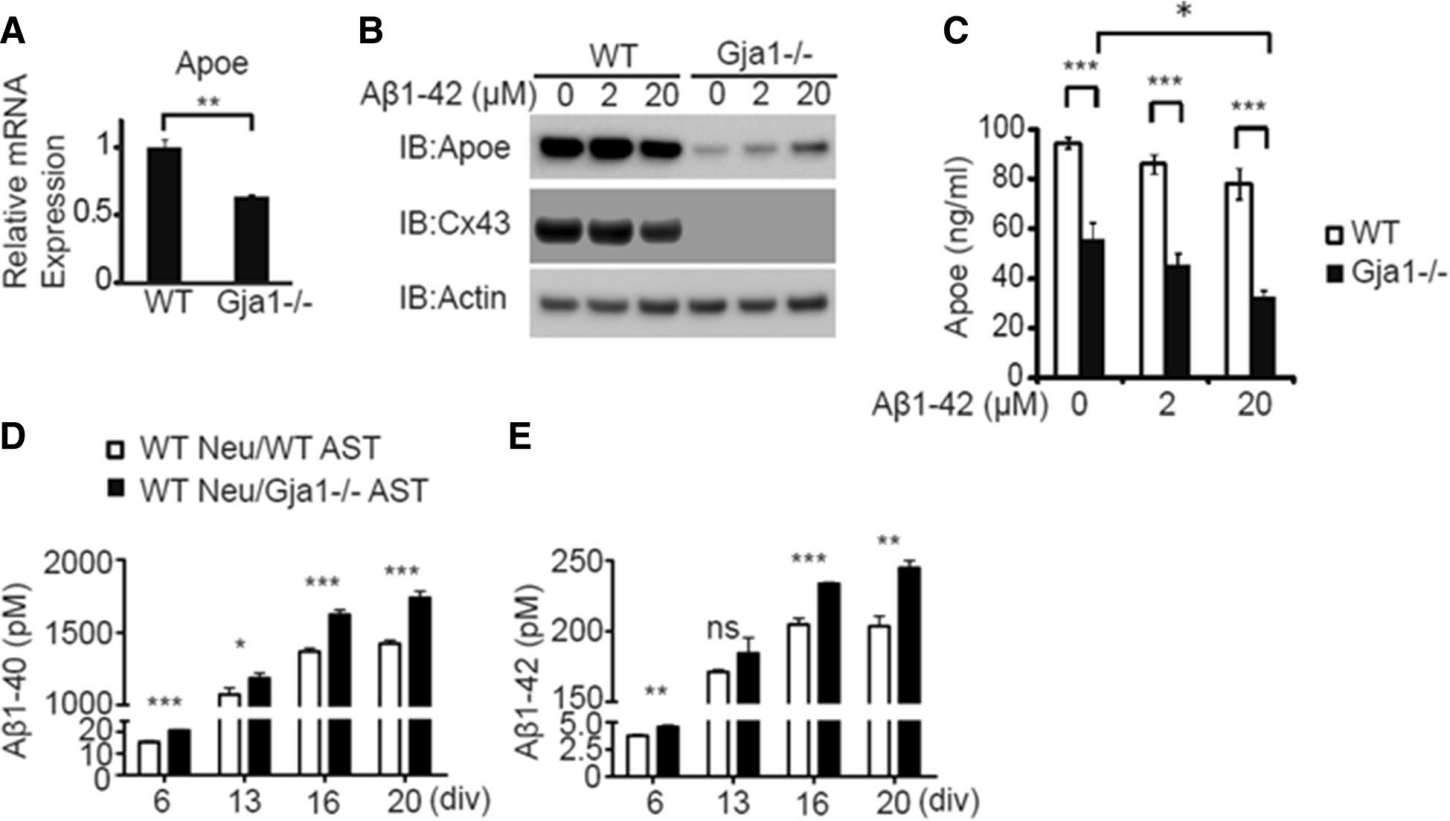
*Gja1* deficiency causes reduction of Apoe production and promotes Aβ production in neuron/astrocyte cocultures. **a**. Quantitative PCR analysis of Apoe in wildtype (WT) and *Gja1−/−* astrocytes. **b**. Apoe, Cx43, and actin levels in wildtype and *Gja1*−/− astrocytes treated with the indicated concentration of *A*β_1–42_ oligomers for 48 h were determined by immunoblot analysis of cell lysates. **c**. Apoe secreted into the conditioned medium of astrocytes in B was quantitatively determined by specific ELISA in quadruplicate wells. **d** and **e**. Amyloidβ secreted to the conditioned medium of wildtype cortical neurons cocultured with either wildtype or *Gja1−/−* astrocytes was quantitatively determined by Aβ_40_ and Aβ_42_ specific ELISA

*APOE* is a well-known risk gene for LOAD and primarily produced by human and mouse astrocytes as well as microglia and neurons [52]. In addition, *APOE* was in the coexpression network module governed by *GJA1*. Therefore, we tested how Apoe levels were affected in the absence of *Gja1* in mouse primary astrocytes. Quantitative PCR revealed significantly reduced *Apoe* expression in *Gja1−/−* astrocytes (t (6) = − 5.330, *p* = 0.002) (Fig. 8a). Consistently, we found lower Apoe protein levels in *Gja1−/−* astrocytes by immunoblot analysis of the lysates, with or without *A*β_1–42_ oligomer treatment (Fig. 8b). We analyzed Apoe in the conditioned medium from these astrocytes and found that secreted Apoe in the conditioned medium was consistently reduced in Gja1−/− astrocytes compared to wildtype, with or without *A*β_1–42_ oligomer treatment (Fig. 8c). Two way ANOVA revealed that there were significant main effects by genotype and treatment (F(1, 18)=121.618, *p* < 0.0005, F(2, 18)=9.364, *p* = 0.0016, respectively) but there was no interaction of genotype and treatment (F(2, 18) = 0.305, *p* = 0.741].

### Neurons cocultured with Gja1−/− astrocytes produce higher levels of Aβ species

Since our RNA-seq analysis revealed that Gja1 regulated genes were highly enriched in Aβ network, we therefore analyzed Aβ production in neuron/astrocyte cocultures. Indeed, we observed higher levels of both Aβ_40_ and Aβ_42_ species in the conditioned medium from neurons co-cultured with *Gja1*−/− astrocytes at all time points examined [Aβ_40_; t(4) = 17.684, *p* < 0.0005 (div6), t(4) = 3.447, *p* = 0.026 (div13), t(4) = 11.112, *p* < 0.0005 (div16), t(4) = 11.112, *p* < 0.0005 (div20): Aβ_42_; t(4) = 8.422, *p* < 0.0005 (div6), t(4) = 2.047, *p* = 0.110 (div13), t(4) = 11.245, *p* < 0.0005 (div16), t(4) = 8.016, *p* < 0.001 (div20)] (Fig. 8d-e). Because astrocytes have been shown to take up *A*β, preferentially oligomers [67], we investigated the role for *Gja1* in controlling Aβ clearance. When we tested whether wildtype and *Gja1*−/− astrocytes were proficient in taking up exogenously applied fluorescently labeled *A*β_1–42_ oligomers, we found that the number of *Gja1*−/− astrocytes with cell-associated *A*β_1–42_ oligomers was significantly reduced compared to wildtype astrocytes [t(20) = − 6.670, *p* < 0.0005] (Additional file 2: Figure S7).

### Reduced neuronal activity in response to Aβ treatment when co-cultured with *Gja1−/−* astrocytes

**Fig. 9:**
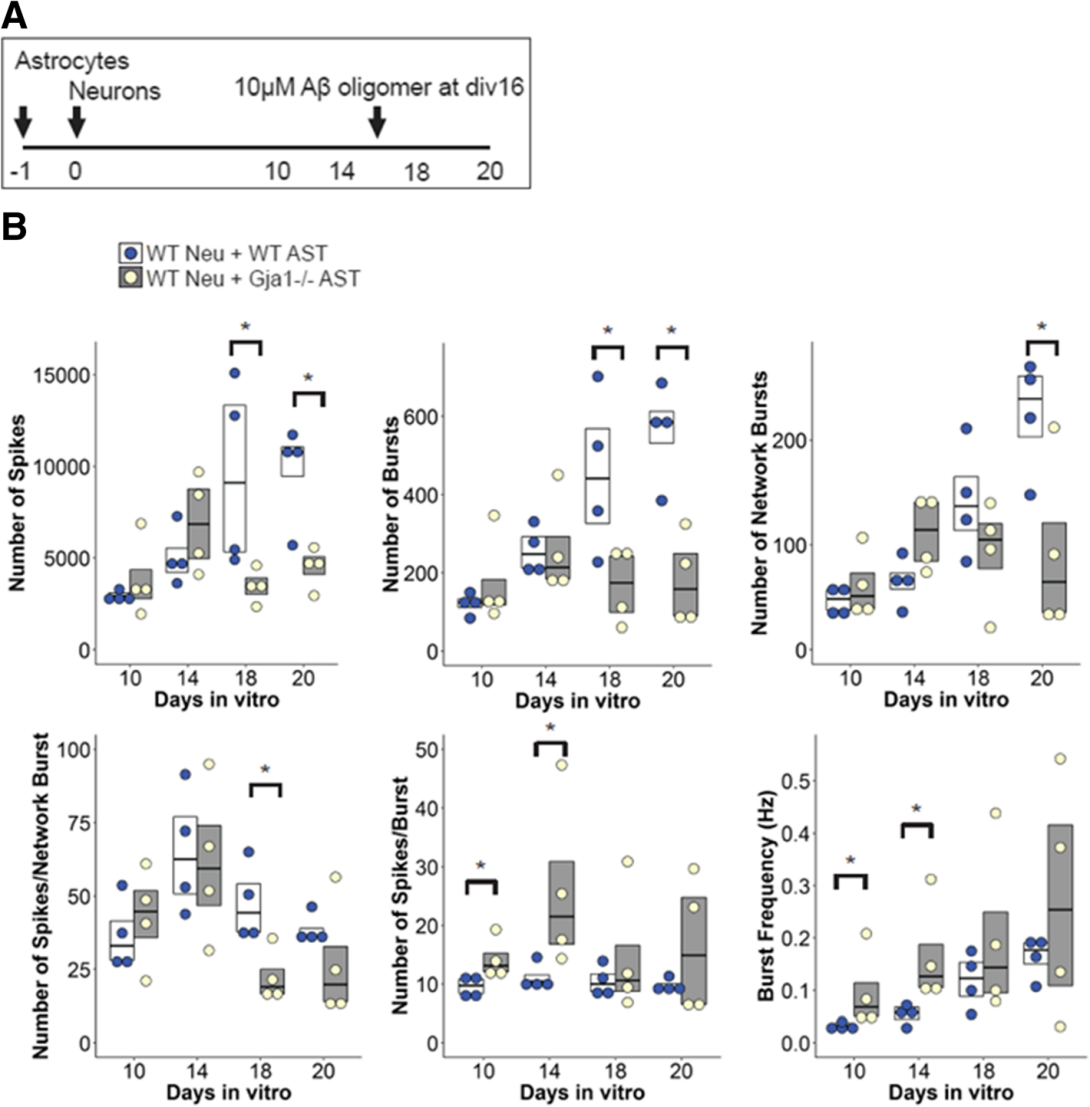
*Gja1* deficiency reduces spontaneous neuronal activity in cortical neuron/astrocyte cocultures as determined by multielectrode array. **a**. Experimental scheme is indicated. One day prior to cortical neuron preparation and plating, wildtype or *Gja1−/−* astrocytes were plated onto 48-well MEA array plate in quadruplicate wells. Spontaneous activities of the cocultures were recorded at day 10, 14, 18 and 20 on Axion Maestro. At day 16, cocultures were treated with 10 μM *A*β_1–42_ oligomers. **b**. Boxplots of the indicated metrics from the MEA recordings are shown. Each circles represent mean values of individual well, and the horizontal bars within the box indicates means of quadruplicates. Mann-Whitney U tests were used for statistical analysis. * *p* < 0.05

**Fig. 10:**
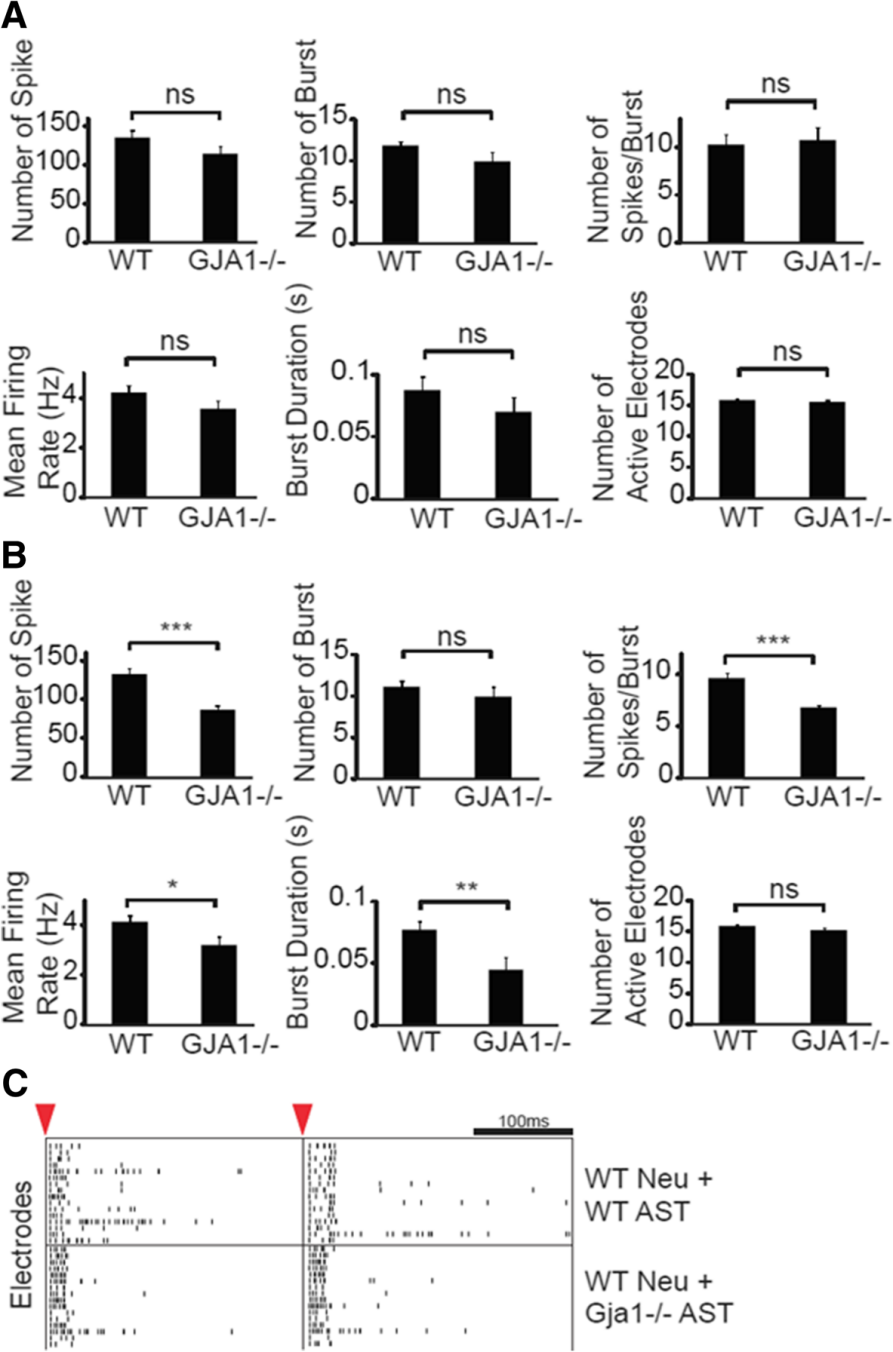
*Gja1* deficiency reduces stimulated neuronal activity in cortical neuron/astrocyte cocultures as determined by multielectrode array. **a** and **b**. Cocultures were stimulated by 3 cycles of voltage-controlled biphasic stimulus from all electrodes within the wells at 14 days (**a**) and 20 days (**b**) in vitro. At least 5 times of repeated stimulations were recorded and analyzed. **c**. Two representative raster plots as recorded on day 20 are shown for wildtype neurons cocultured with wildtype astrocytes (upper panel) or with *Gja1−/−* astrocytes (lower panel). Y-axis represents 16 electrodes and X-axis represents time after stimulation indicated by red arrowhead. Black horizontal scale bar indicates 100 milliseconds (ms). Each vertical bar represents a detected spike

In order to understand the impact of the network perturbation by loss of astrocytic *Gja1*−/− on neuronal functions, we co-cultured wildtype cortical neurons with either wildtype or *Gja1*−/− astrocytes on multielectrode arrays and compared spontaneous and stimulated neuronal activities before and after *A*β_1–42_ oligomer treatment (Fig. 9a). Although few significant differences were detected prior to Aβ treatment at 16 days in vitro (div), at 18 and/or 20 div (2 and 4 days after 10 μM *A*β_1–42_ treatment) most metrics of spontaneous activity (i.e. number of spikes, bursts, network bursts, and spikes per network burst) were significantly reduced in *Gja1*−/− astrocyte-cocultured neurons (Fig. 9b). The reduced spontaneous neuronal activity at div 20 suggested that co-culture with *Gja1−/−* astrocytes made neurons less responsive to Aβ-induced changes. While stimulated neuronal activity at div 14 was not affected by co-culture with *Gja1−/−* astrocytes (Fig. 10a), it was reduced at 20 div (number of spikes, spikes per burst, mean firing rate, and burst duration) (Fig. 10b-c).

## Discussion

Functional interaction between neurons and astrocytes is an appealing research area that has expanded vigorously in recent years. The involvement of astrocytes in the pathogenesis of neurodegenerative diseases such as AD is just beginning to be appreciated. Astrocyte-specific *GJA1* expression has been shown to be upregulated in the postmortem AD brains [6, 34]. Consistent with but beyond these studies, we predicted and validated not only upregualtion of *GJA1* expression in AD but also its regulatory role in driving a large astrocyte-specific molecular network underlying AD. Indeed, our functional analysis using *Gja1−/−* astrocytes revealed that the *Gja1* network contributed to neuronal death and decreased their viability under *A*β_1–42_ treatment and, which may explain synapse and neuron loss in the Alzheimer brains [20]. This is consistent with recent studies showing that *GJA1* play critical roles in AD-relevant phenotypes in AD mouse models [40, 73, 96, 97]. On the other hand, a number of other studies indicate that *Gja1* isneuroprotective [41, 44, 51, 64, 65, 85]. Indeed, our data showed that the *Gja1* network appeared to be involved in phagocytosis of Aβ, and maintenance of neuronal activities under Aβ stress. Taken together with the previous findings of the increase of Cx43 protein and its channel activity specifically in astrocytes surrounding plaques [59, 63, 96], we suggest that the upregulation of *GJA1* in AD brains may start as a neuroprotective response to amyloid plaques. It is interesting to note that previous clinical studies have consistently demonstrated an increased neuronal activity in the hippocampus and cortex in non-demented individuals at high risk for AD including *APOE4* carriers, minor cognitive impairment (MCI) or at presymptomatic stage of familial Alzheimer’s disease [10, 72], and individuals with early Alzheimer’s disease are prone to developing seizures [87]. Furthermore, the hippocampal hyperactivation is a viable therapeutic target for AD [4]. We speculate that the role of *GJA1* in supporting neuronal activity in the AD brain might explain partly the observation of elevated brain activity in the prodromal stage of AD.

In the present study, we demonstrated *GJA1* as a master regulator, which regulates not an AD-related astrocyte-specific gene subnetwork [98] but also an array of AD risk genes including the most important AD gene *APOE* (Fig. 4a-b). More broadly speaking, in in vitro cultured astrocytes, *Gja1* deficiency caused significant change in expression of 5496 genes involved a diverse of biological pathways and processes (Fig. 3b). Similarly, in in vitro astrocyte and neuron cocultures, thousands of genes were regulated by Gja1 (−/−). Of note, more than half of the known AD risk factor genes (Fig. 2g) and hundreds of inflammatome genes in chronic inflammation diseases [23] were the targets of *GJA1*. Upregulation of *GJA1* expression in AD brains of human subjects and in vitro or in vivo perturbation of *Gja1* (e.g., *Gja1* deficiency in primary cultured astrocytes in the present study) lead to a cascade of biological and pathological reactions and processes including subsequent alteration in cells’ biological functions and responses to their environments, which are manifested as a complex and intertwined *GJA1* centered molecular network. Interestingly, our data pointed to the key role of Cx43 gap junction channel and hemichannel activities in regulating gene networks, and indicated that the network can be fine-tuned by pharmacologically modulating Cx43 channel activity (Fig. 6 and Additional file 2: Figures S5 and S8). Cx43 could be a useful therapeutic target in diseases such as LOAD, ODDD, and other neuropsychiatric disorders [71]. In support for such a hypothesis, recent studies showed that *Gja1*-deficiency or a connexin inhibitor (Boldine) treatment in APP/PS1 mice had beneficial effects [73, 96, 97], underscoring that Cx43 is a critical mediator of AD pathophysiology and an important therapeutic target for LOAD.

One of the important novel discoveries in the present study was the *Gja1* mediated production of ApoE in astrocytes. APOE is mainly produced in astrocytes, but also in neurons and microglia when under stress [35]. APOE is the major LOAD risk factor, critical to AD neuropathology and neurocognitions [15, 39]. We therefore focused on characterizing Apoe in *Gja1−/−* astrocytes. Manipulation of *Gja1* by genetic disruption, cytokines, CBX, and quinine in astrocytes as well as neuron/astrocyte/microglia culture system consistently resulted in the regulation of Apoe in the same direction as *Gja1*. Abnormal production of APOE, in particular, the risk-associated isoform APOE4, caused either gain of toxic functions, when over-expressed, such as neuronal toxicity, Aβ aggregation and tangle formation or loss of physiological functions, when under-expressed, such as Aβ clearance, synaptic function and neurogenesis. Consistent with this notion, we observed drastic reduction in uptake and retention of Aβ oligomers by Gja1(−/−) astrocytes (Additional file 2: Figure S7 A-B), with concomitant increase in concentration of both Aβ1–40 and Aβ1–42 species in in vitro culture medium (Fig. 8d-e). Since *APOE* is known to be regulated by transcription factors such as *LXR* and *RXR* [48], and taken together with downregulation of *NR1H3* (LXRA) expression in our RNA-seq data, we speculate that *NR1H3* regulation by *GJA1* might have mediated the *APOE* expression. Further investigation is required to dissect the mechanism underlying GJA1-mediated expression of APOE and its biological significance, which would lead to the development of novel therapeutic strategies.

A recent study identified the transcriptomic basis of the two distinct polarization states of A1 neurotoxic and A2 neuroprotective astrocytes [50]. Interestingly, our RNA-seq analysis revealed that most of pan-reactive, A1 and A2 specific transcripts were significantly upregulated in *Gja1*−/− astrocytes (Additional file 2: Figure S5). This pattern of expression is most similar to those induced by combination of IL-1β and TNFα or C1q (Fig. 1a in [50]), and consistent with this, our data showed that IL-1β and TNFα caused downregulation of Cx43 and other key drivers, mimicking the loss of *Gja1*. This pattern of expression may indicate that *Gja1* deficiency induces non-specific activation of either A1 or A2 polarized-astrocytes, or A1/A2 overlapping polarization. Although these two possibilities need to be clarified by transcriptomic analysis at a single cell level, these mixed transcriptomic signatures suggest how *Gja1* may contribute to opposing neuroprotective (Figs. 8, 9 and 10) and neurotoxic (Fig. 7) phenotypes, as demonstrated here as well as in previous studies [41, 44, 51, 64, 65, 69, 85, 96, 97].

**Fig. 11:**
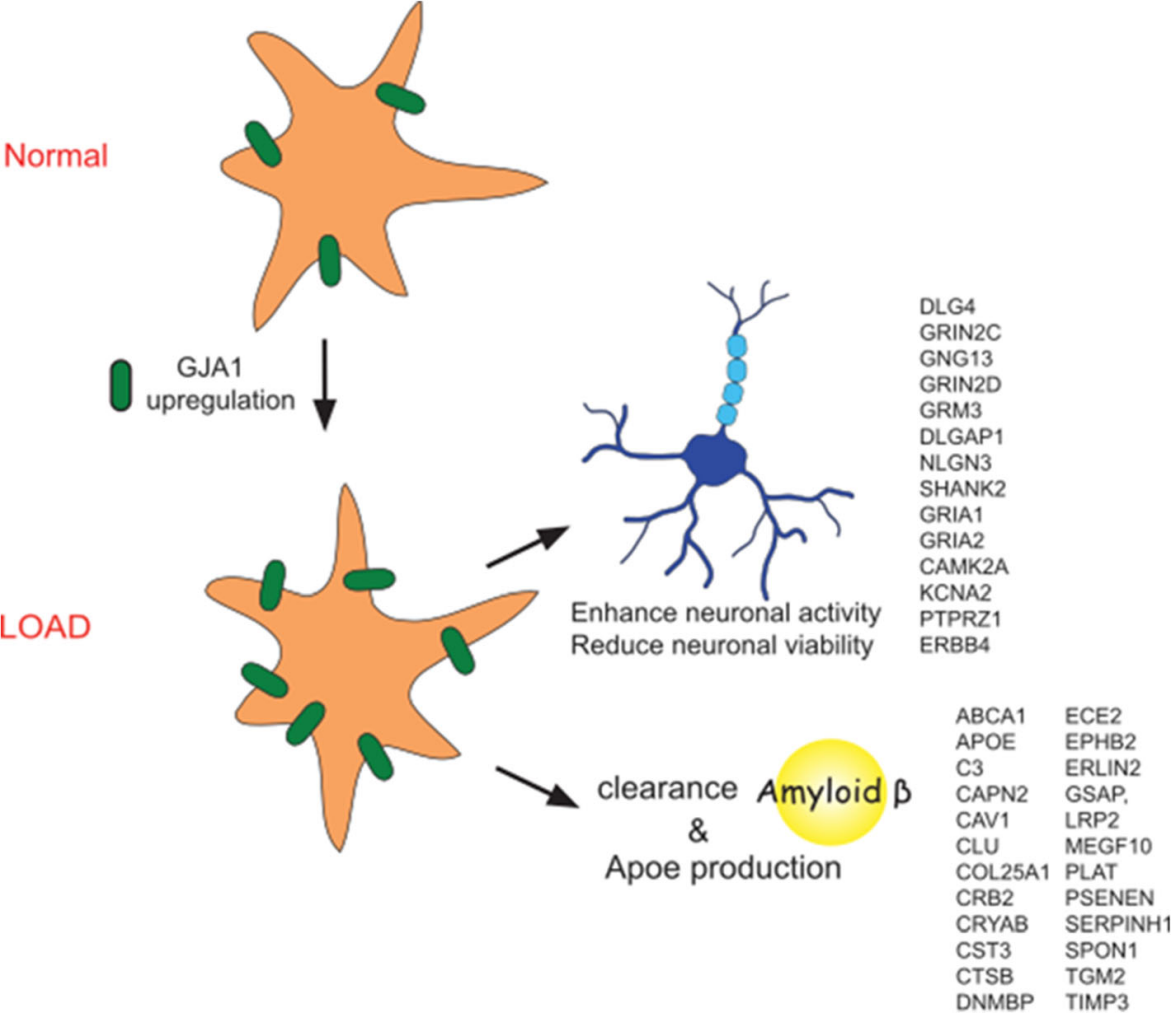
A working hypothesis of *GJA1* dysregulation in AD. Progressive upregulation of *GJA1* in the brains during LOAD pathogenesis (in response to amyloid accumulation) serves to enhance the coordinated gene network function to orchestrate neuroprotective response by astrocytes including Aβ production and clearance (*CST3, CLU, CTSB, ECE2, MEGF10, PSENEN* and *GSAP*), APOE production (*APOE*, *ABCA1, CAV1* and *SPON1*), and supporting neuronal activity (*DLG4, NLGN3, GRINA* and *GRIN2D*). However, the enhanced neuronal activity due to chronic and prolonged *GJA1* upregulation triggers neuronal wear and eventual death

Based upon the results from this study and the discussion above, we developed a working hypothesis of *GJA1* dysregulation in AD (Fig. 11), in which upregulation of *GJA1*, a master regulator of astrocytic gene expression, concomitant with amyloid accumulation during Alzheimer pathogenesis ultimately drives AD clinical and pathological traits as evidenced by highly significant correlation with AD progression and AD GWAS genes (Fig. 2a-g). According to our model, *GJA1* induction initially serves to support neuronal functions by elevating gene expression signatures for the glial neurosupportive functions such as Abeta network (Fig. 3c), Apoe (Fig. 8a-c), vascular and neurodevelopment, extracellular matrix, and cytoskeletal and cell adhesion (Fig. 3a-b) as well as suppressing inflammatory response (Fig. 3b). These findings were supported by amyloid phagocytosis (Fig. 8d-e, Additional file 2: Figure S5) and multielectrode array (Figs. 9 and 10) assays showing that the amyloid uptake, Apoe expression, and neuronal activity are attenuated with Gja1−/− astrocytes. This is consistent with several key clinical and pathological observations in LOAD. For example, neuronal activity is found to be enhanced at the early stage of Alzheimer’s disease subjects [4, 10, 72, 87] and evidence of cell cycle activation in AD neurons [3, 53, 58, 79, 86, 94], changes in extracellular matrix [12, 46], and neuronal cytoskeletal components [5, 13, 45, 91] have been reported. However, we speculate that chronic *GJA1* gain of function could lead to detrimental effects on neurons in a long run, as evidenced by our observation, in which prolonged neuron/astrocyte cocultures were better protected in the absence of *Gja1* in astrocytes (Fig. 7a-b) possibly due to excessive neuronal activity and glutamate-induced excitotoxicity [24, 37, 47, 68].

In summary, this study elucidated the previously unrecognized role for *GJA1* and its channel activity in regulating a key AD-related gene regulatory network comprised of *APOE* and other astrocyte-specific genes. We demonstrated that the disruption of this astrocytic gene coexpression network by knocking out *Gja1* can contribute to LOAD-related pathology. This study represents the first step towards a comprehensive functional characterization of one of the most significantly dysregulated modules in our previous integrative network analysis of LOAD brains. The strong validation of the predictive power of our multiscale gene network approach paves a way to dissect the complex pathogenic mechanisms of LOAD.

## Additional files

Additional file 1: **Tables S1.** Clinical and pathological traits, brain regions analyzed by transcriptome profiling, and sample classification with respect to the severity stage of each trait. **Table S2.** Correlations between GJA1 expression and individual phenotypical traits. **Table S3.** Differential expression analysis. **TableS4.** Covariables' impacts on GJA1 association with AD traits. **Table S5.** Gene ontology analysis. **Table S6.** Intersection of GJA1 gene signatures and AD gene signatures. **Table S7.** Intersection of GJA1 gene signatures and the khiki module. **Table S8.** GJA1_centered genetic network analysis. **Table S9A.** GJA1 DEG signatures (AST+NEU) overlapped with brain cell type specific gene signatures. **Table S9B.** AST+NEU vs AST in Gja1 (-/-) or (+/+) DEG signatures overlapped with brain cell type specific gene signatures. **Table S9C.** differentially expressed genes in neurons upon GJA1 KO or not in astrocyte and neuron coculture. **Table S9D.** Difference of change degree in AST+NEU_vs_AST_Gja1(-/-) vs AST+NEU_vs_AST_Gja1(+/+). **Table S10.** List of primer sets and Universal Probe Library (UPL) probe for qRT-PCR. (XLSX 234 kb)

Additional file 2: **Figure S1.** Correlations between GIA1 and individual clinic traits stratified by AOD (age of death), gender and APOE genotypes in BM36 region. **Figure S2.** Correlation between Gja1 and individual transcripts of ADGWAS genes. **Figure S3.** GJA1-centric Bayesian causal network and GJA1 signaling pathway map. **Figure S4.** Enrichment of Gia1-/- gene signatures in GJA1 centric correlation networks. **Figure S5.** Regulation of A1/A2 astrocyte marker genes in Gja1-/- astrocytes. **Figure S6.** Quinine (GJA1 channel agonist) upregulates Gja1 and Apoe, and other network genes. **Figure S7.** A. Wildtype and Gja1-/- astrocytes were treated with fluorescently labeled Aβ1-42 oligomers for 24 hours and the number of astrocytes in association with Aβ1-42 oligomers were quantitatively estimated by counting total cells (DAPI+) and Aβ positive cells. B. Representative images of phase contrast, Hilyte Fluor488-labeled Aβ, and DAPI staining from wildtype (upper panels) and Gja1-/- (lower panels) astrocytes were shown. These images were taken by bright field microscope and the representatives of 2 independent experiments with similar results are shown. **Figure S8.** Complex regulation of Gja1 key drivers in neuron/astrocyte/microglia cocultures by carbenoxolone and quinine. (DOCX 1.4 kb)
