# Supplementary material for: GJA1 (connexin43) is a key regulator of Alzheimer’s disease pathogenesis
Source: Acta Neuropathol Commun. 2018 Dec 21;6:144. doi: 10.1186/s40478-018-0642-x (PMC6303945; doi:10.1186/s40478-018-0642-x)
Supplement: Supplementary file 2 — Figure S1. Correlations between GIA1 and individual clinic traits stratified by AOD (age of death), gender and APOE genotypes in BM36 region. Figure S2. Correlation between Gja1 and individual transcripts of ADGWAS genes. Figure S3. GJA1-centric Bayesian causal network and GJA1 signaling pathway map. Figure S4. Enrichment of Gia1-/- gene signatures in GJA1 centric correlation networks. Figure S5. Regulation of A1/A2 astrocyte marker genes in Gja1-/- astrocytes. Figure S6. Quinine (GJA1 channel agonist) upregulates Gja1 and Apoe, and other network genes. Figure S7. A. Wildtype and Gja1-/- astrocytes were treated with fluorescently labeled Aβ1-42 oligomers for 24 hours and the number of astrocytes in association with Aβ1-42 oligomers were quantitatively estimated by counting total cells (DAPI+) and Aβ positive cells. B. Representative images of phase contrast, Hilyte Fluor488-labeled Aβ, and DAPI staining from wildtype (upper panels) and Gja1-/- (lower panels) astrocytes were shown. These images were taken by bright field microscope and the representatives of 2 independent experiments with similar results are shown. Figure S8. Complex regulation of Gja1 key drivers in neuron/astrocyte/microglia cocultures by carbenoxolone and quinine. (DOCX 1.4 kb) [file 40478_2018_642_MOESM2_ESM.docx]

**SUPPLEMENTARY MATERIALS**

**Supplementary Methods**

**Transcriptomic Datasets**

To gain insight into the roles *GJA1* may play on AD pathology and cognitive functions, we first extensively investigated how *GJA1* expression at the mRNA level was correlated with AD neuropathological traits in 32 gene expression datasets from three AD cohort studies of aging and dementia that included organ donation at death: the Mount Sinai/JJ Peters VA Medical Center Brain Bank (MSBB) ^36^, the Religious Orders Study and the Rush Memory and Aging Project (ROSMAP) ^37-38^ and in the Harvard Brain Tissue Bank Alzheimer’s Disease study (HBBAD) ^1^. We chose six different clinical and pathological criteria to evaluate the clinical relevance of *GJA1* on AD pathology and cognitive functions: the MiniMental State Examination (MMSE) score ^39-40^, the sum of NFT density estimates for all cortical regions examined (NTrSum), Mean Plaque density (PLQ_Mn) for the estimation of average plaque density, Braak stage score for quantitative assessment of neurofibrillary tangles ^41^, the Consortium to Establish a Registry for Alzheimer’s disease (CERAD) score for quantitative measure of neuritic plaques, and clinical dementia rating score (CDR).

***GJA1* expression is associated with AD clinical and pathophysiological traits**

In the ROSMAP cohort, *GJA1* expression in the microarray was significantly correlated with CERAD score (r = -0.1516, p = 0.0033) and the MiniMental State Examination (MMSE) score (r = - 0.1407, p = 0.0064), and similar results were observed in the ROSMAP RNA-seq data (**Figure 2, and Additional file 1: Table S2**), suggesting that the mRNA expression of *GJA1* is associated with AD pathogenesis and dementia.

The MSBB AD cohort includes microarray and RNA-seq data from a battery of distinct brain cortical regions and thus provides an excellent opportunity to investigate regional differences in the correlation between *GJA1* expression and AD neuropathological traits ^36^. Among the 19 brain cortex regions investigated in the MSBBAD microarray data, *GJA1* had a significant correlation with at least three of the five AD neuropathological traits in the six cortex regions (BM10 (frontal pole), BM20 (inferior temporal gyrus), BM21 (middle temporal gyrus), BM32 (anterior cingulate), BM36 (parahippocampal gyrus), and BM46 (dorsolateral prefrontal cortex)) (**Figure 2, and Additional file 1: Table S2**). Overall, *GJA1* expression in these six cortex regions displayed a significant positive correlation with Braak stage score, PLQ_Mn, NTrSum and CDR. The MSBBAD RNA-seq assays revealed a consistent pattern of correlation between *GJA1* expression and AD clinic traits across the cortex regions studied **(Additional file 1: Table S2**). Notably, in BM10, BM36 and BM44 cortex regions, microarray and RNA-seq assays converged to show a consistent correlation between *GJA1* expression and AD neuropathological traits (**Additional file 1: Table S2**). Thus, in the MSBB cohort, the association between *GJA1* expression with AD neuropathological traits was cortex-specific. At the protein level, GJA1 in the brain cortex BM10 region was significantly correlated with CERAD (r= -0.317, p = 1.26E-07), PLQ_Mn (r = 0.371, p = 4.08E-10) (**Figure 2**) and CDR (r = 0.347, p = 5.69E -9) (**Additional file 1: Table S2**). Strikingly, total soluble amyloidβ (Aβ) level also had a significant positive correlation with GJA1 protein level in BM10 region (r= 0.178, p= 0.0036).

Taken together, a significant correlation exists between *GJA1* at mRNA and protein levels in the cortex and the AD neuropathological traits that are involved in major aspects of AD pathology and cognitive functions.

***GJA1* is up-regulated in AD**

To further determine whether a causal relationship existed between *GJA1* expression, and AD pathology and cognitive functions, we performed pairwise Student’s t-tests to compare levels of *GJA1* expression across a range of AD neuropathological traits. To achieve this, we stratified individual cohort datasets into three classes/groups with low, medium and high severity of AD symptoms based on each individual AD neuropathological and functional cognitive traits according to the criteria established in the ROSMAP clinic codebook (**Additional file 1: Table S1**). As described above, we selected six individual AD clinical traits (CDR, MMSE, CERAD, PLQ_Mn, Braak, and NTrSum, if applicable in the cohorts under study) to compare the difference in *GJA1* average expression over groups of individual neuropathological traits.

In the ROSMAP cohort, a significant difference in *GJA1* expression was detected in high vs low groups of each of the three AD neuropathological traits, Braak score, CERAD, and MMSE as assessed by both microarray and RNA-seq assays.

In the MSBB cohort, among the 19 brain regions evaluated by microarray assays, BM10 had at least one significant change in *GJA1* expression in the three group comparisons (medium vs. low, high vs. medium, and high vs. low) across all the AD neuropathological traits assessed; Amygdala (AMYG), Inferior Temporal Gyrus (BM20), Middle Temporal Gyrus (BM21), Superior Temporal Gyrus (BM22), Anterior Cingulate (BM32), Dorsolateral Prefrontal Cortex (BM46), Superior Parietal Lobule (BM7), Nucleus Accumbens (NAC) and Putamen (PT) regions had at least one significant change in *GJA1* expression in the three group comparisons in two or more of the AD neuropathological traits assessed; BM36, Temporal Pole (BM38), Inferior Frontal Gyrus (BM44), Superior Frontal Gyrus (BM8) and Hippocampus (HIPP) regions had at least one significant change in *GJA1* expression in the three group comparisons in one of the AD neuropathological traits assessed; and no significant change in *GJA1* expression was observed for the remaining brain regions (Additional file 1: Table S2). Among the four brain regions evaluated by RNA-seq assays, BM36 had at least one significant change in *GJA1* expression in the three group comparisons (medium vs. low, high vs. medium, and high vs. low) across all the AD neuropathological traits assessed whereas BM10, BM22 and BM44 had at least one significant change in *GJA1* expression in the three group comparisons across most of the AD neuropathological traits except for Braak score (BM22 and BM44) or CERAD (BM10) (**Additional file 1: Table S3**). Further, Cx43 protein levels in BM10 possessed at least one significant difference among the three group comparisons (medium vs. low, high vs. medium, and high vs. low) across the AD neuropathological traits assessed (CDR, PLQ_Mn, and CERAD; **Additional file 1: Table S3**).

*GJA1* demonstrated a causal relationship with AD neuropathological traits and cognitive functions, with low *GJA1* levels in normal controls that was increased as the severity of AD symptoms increases. Correlation and differential expression analysis converged to demonstrate that *GJA1* was relevant to both amyloid and tau pathologies of AD, and furthermore, that *GJA1* impacted cognitive functions, suggesting that *GJA1* may play an important role in AD.

**Supplementary Figures (8, in total)**


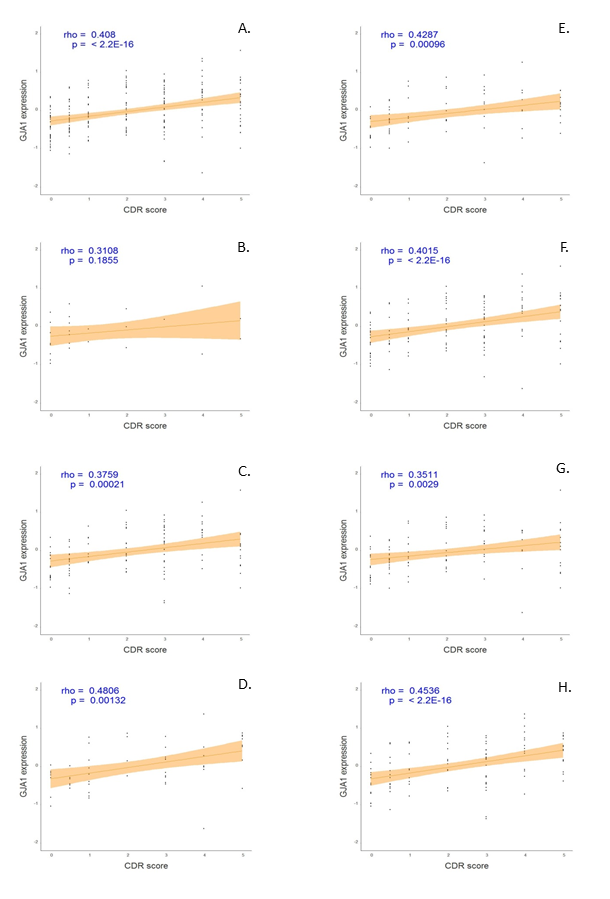


**Figure S1. Correlations between *GIA1* and individual clinic traits stratified by AOD (age of death), gender and APOE genotypes in BM36 region.** A. All subjects included without stratification; B. APOE23; C. APOE33 D. APOE34; E. male; F. female; G.AOD < 85; and H. AOD >= 85.


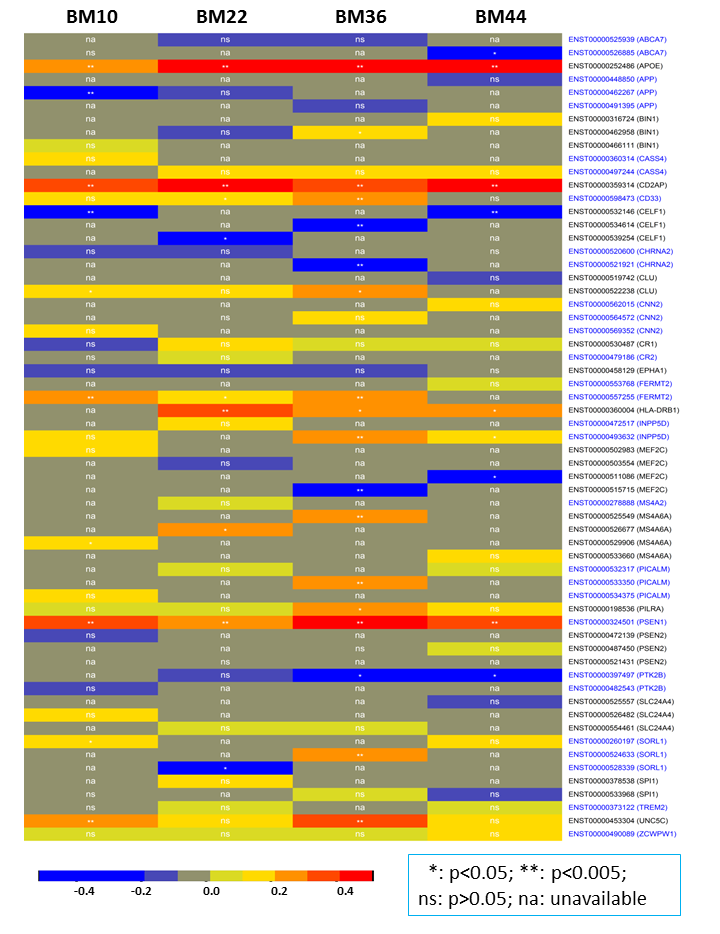
**Figure S2. Correlation between Gja1 and individual transcripts of ADGWAS genes.** **, p < 0.005; *, p < 0.05; ns, not significant; and na, not applicable, RNA-seq reads are below detection.

**
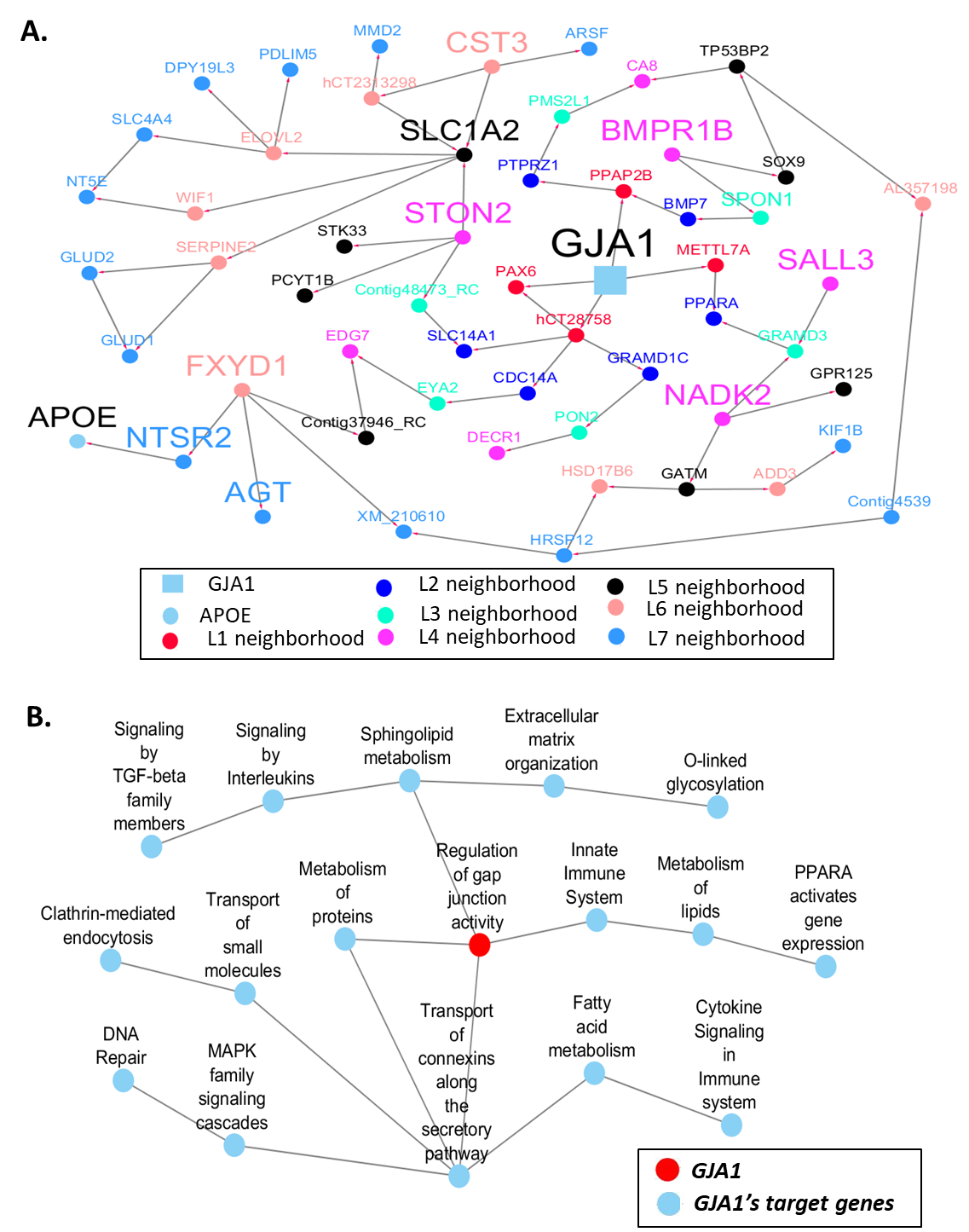
**

**Figure S3. *GJA1*-centric Bayesian causal network and *GJA1* signaling pathway map. A.** A Bayesian causal network regulated by *GJA1*. The labels of the genes validated by the *in vitro* experiments are shown in a larger font size. **B.** *GJA1* signaling pathway map. This signaling map was constructed by replacing each individual node in the Bayesian causal network with the most enriched gene ontology in each node’s direct neighborhood.

**
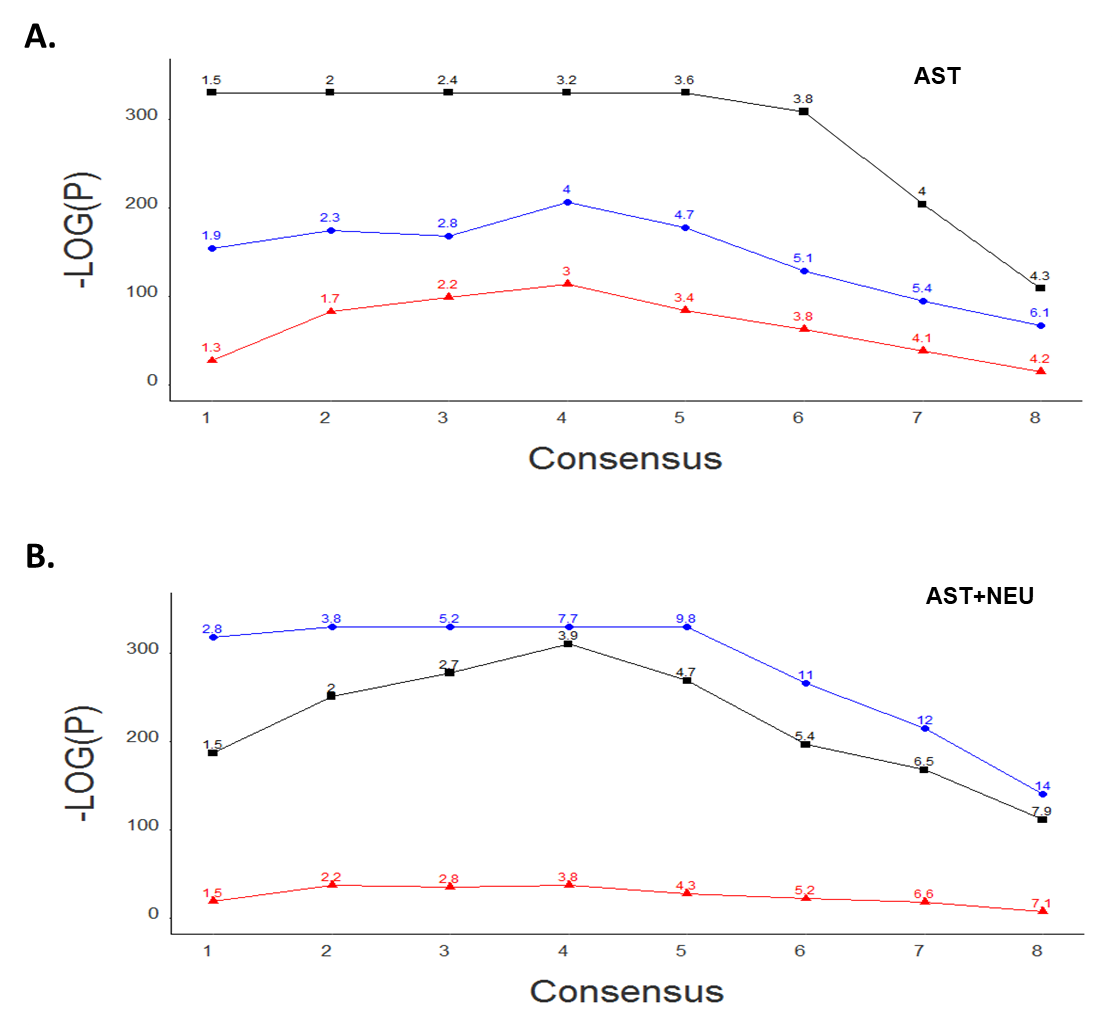
**

**Figure S4. Enrichment of *Gia1-/-* gene signatures in *GJA1* centric correlation networks.** The DEG signatures were identified between *Gja1*-/- and wildtype astrocytes (**A**) and between coculture of *Gja1*-/- astrocytes and wildtype neurons and that of coculture of *wildtype* astrocytes and wildtype neurons (**B**). The two *Gja1*-/- signatures were projected onto the consensus *GJA1*-centric correlation signatures (CGCCS(n), n=1, 2, …, 8), The y-axis is the –log10 (p-value) from Fisher’s Exact Test (FET) of *Gja1-/-* DEG signatures and the CGCCS signatures. Blue, red and black lines are the enrichment for the down-regulated genes, the up-regulated genes and all the genes in a CGCCS signature. Numbers on the lines are the corresponding fold enrichment.

**
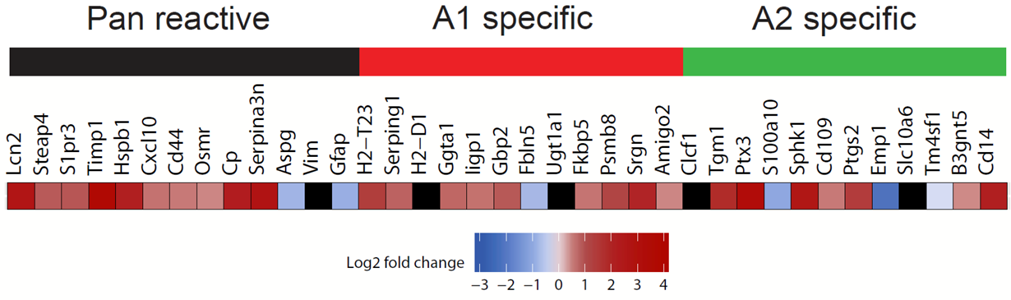
**

**Figure S5. Regulation of A1/A2 astrocyte marker genes in *Gja1*-/- astrocytes.** Summary of RNA-seq results comparing wildtype and *Gja1*-/- astrocytes for the pan reactive, A1 and A2 specific astrocyte genes identified in ^46^ are shown. Red and blue fill indicate significant upregulation and downregulation in *Gja1*-/- astrocytes, respectively. Black fill indicates that the genes are not significantly altered.

**
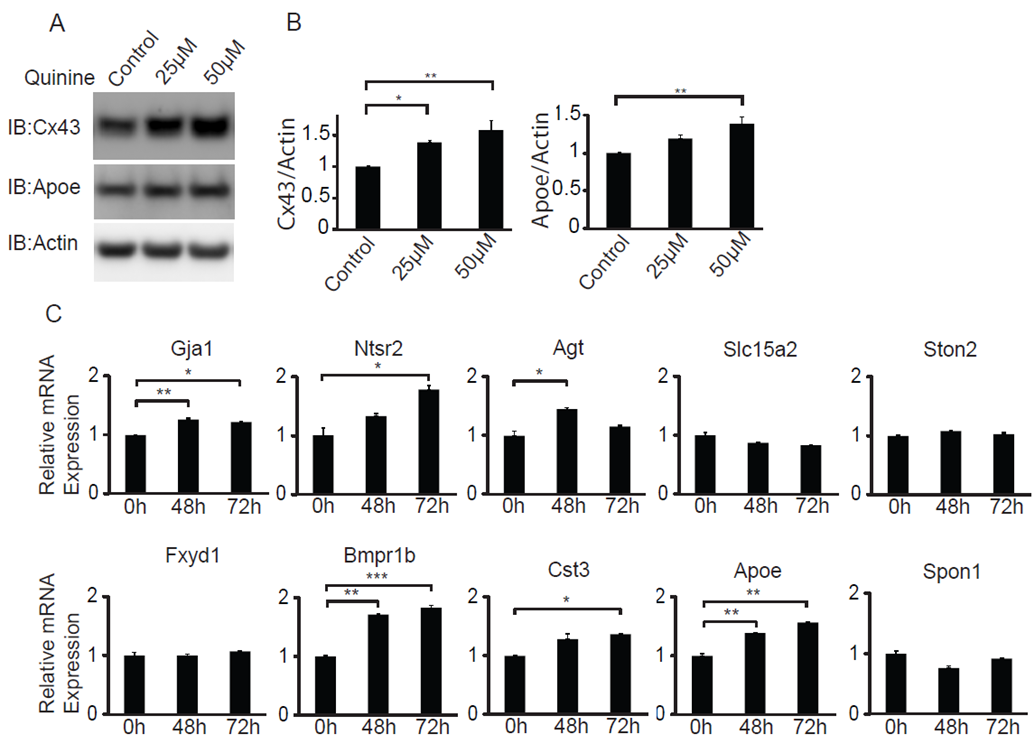
**

**Figure S6. Quinine (*GJA1* channel agonist) upregulates *Gja1* and Apoe, and other network genes. A.** Wildtype primary mouse astrocytes were treated with 25 or 50 μM quinine for 3 days and levels of Cx43, Apoe were analyzed by immunoblot. Representative results from 2 independent experiments are shown. B. Quantitative densitometry analysis of Cx43 and Apoe protein levels were performed after normalization to Actin. ANOVA followed by Bonferroni post-hoc tests are indicated by asterisks. * p < 0.05, ** p < 0.01. C. Quantitative gene expression analysis in wildtype astrocytes treated with 25 μM quinine for 48 and 72 hours was performed to analyze the other drivers of the khaki module based causal network. Results representative of two independent experiments are shown. Statistical analysis was performed as in B, and only the comparisons to 0 hour are shown.

**
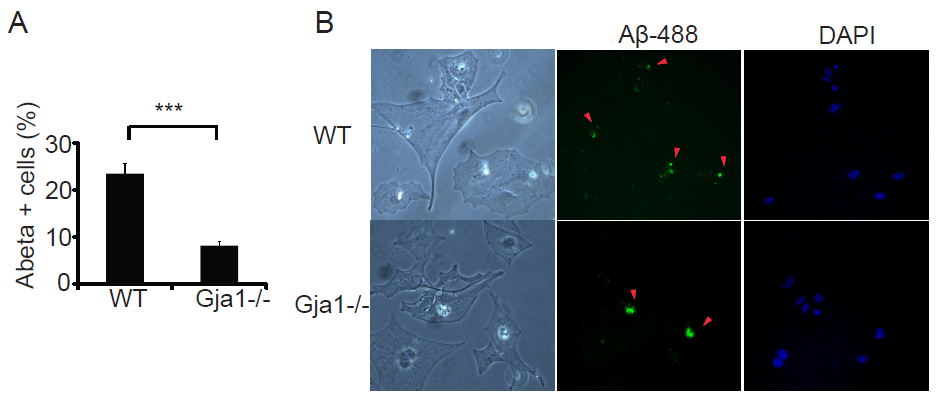
**

**Figure S7**. **A**. Wildtype and *Gja1-/-* astrocytes were treated with fluorescently labeled *A*β_1-42_ oligomers for 24 hours and the number of astrocytes in association with *A*β_1-42_ oligomers were quantitatively estimated by counting total cells (DAPI+) and Aβ positive cells. **B**. Representative images of phase contrast, Hilyte Fluor488-labeled Aβ, and DAPI staining from wildtype (upper panels) and *Gja1-/-* (lower panels) astrocytes were shown. These images were taken by bright field microscope and the representatives of 2 independent experiments with similar results are shown.

**
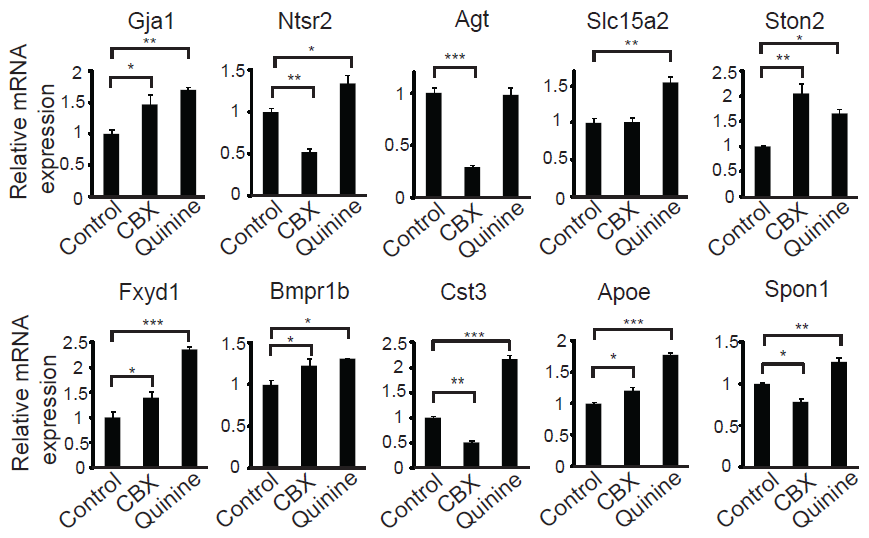
**

**Figure S8. Complex regulation of *Gja1* key drivers in neuron/astrocyte/microglia cocultures by carbenoxolone and quinine.** Fetal cortical neurons (0.5 x 10^6^/well), were plated in 6-well plate and astrocytes (0.5 x 10^6^/well) and microglia (0.1 x 10^6^/well) were subsequently added at 7 and 14 days in vitro, respectively. Cocultures were treated with either control, 200 μM CBX or 25 μM quinine for 3 days. Total RNAs were extracted from 3 replicate wells for each treatment, and gene expression levels of *Gja1* and the key drivers were quantitatively analyzed by qPCR. Student's t-tests were performed between control and either CBX or quinine. * p < 0.05, ** p < 0.01, *** p < 0.001.
